# Clinical Characteristics and Outcomes in Intra-aortic Balloon Pump–Supported Cardiogenic Shock Among Patients Transferred to Tertiary Care Centers

**DOI:** 10.1016/j.jscai.2026.104268

**Published:** 2026-04-07

**Authors:** Jonas Sundermeyer, Rachna Kataria, A. Reshad Garan, Song Li, Van-Khue Ton, Elric Zweck, Manreet K. Kanwar, Jaime Hernandez-Montfort, Shashank S. Sinha, Jacob Abraham, Kevin J. John, Paavni Sangal, Claudius Mahr, Daniel Burkhoff, Navin K. Kapur, Cardiogenic Shock Working Group Academic Research Consortium, Jonas Sundermeyer, Song Li, Van-Khue Ton, Rachna Kataria, Elric Zweck, Kevin J. John, Manreet K. Kanwar, Jaime Hernandez-Montfort, Shashank S. Sinha, A. Reshad Garan, Jacob Abraham, Vanessa Blumer, Ajar Kochar, Karthikeyan Ranganathan, Gavin W. Hickey, Mohit Pahuja, Scott Lundgren, Sandeep Nathan, Esther Vorovich, Shelley Hall, Wissam Khalife, Andrew Schwartzman, Ju Kim, Oleg Alec Vishnevsky, Justin Fried, Maryjane Farr, Joseph Mishkin, I-Hui Chang, Onyedika Ilonze, Alexandra Arias, Jun Nakata, Jeffrey Marbach, Hiram Bezerra, Ann Gage, Joyce Wald, Sunu Thomas, Faisal Rahman, Amirali Masoumi, Aasim Afzal, Salman Gohar, Rachel Goodman, Karol D. Walec, Peter S. Natov, Borui Li, Paavni Sangal, Qiuyue Kong, Peter Zazzali, Neil M. Harwani, Saraschandra Vallabhajosyula, Arvind Bhimaraj, Claudius Mahr, Daniel Burkhoff, Navin K. Kapur

**Affiliations:** n The Cardiovascular Center, Tufts Medical Center, Boston, Massachusetts; o Department of Cardiology, University Heart and Vascular Center Hamburg, University Medical Center Hamburg-Eppendorf, Hamburg, Germany; p Institute for Advanced Cardiac Care, Medical City Healthcare, Dallas, Texas; q Massachusetts General Hospital, Boston, Massachusetts; r Brown University Health Cardiovascular Institute, Providence, Rhode Island; s Department of Cardiology, Pulmonology, and Vascular Medicine, Medical Faculty and University Hospital Düsseldorf, Heinrich-Heine-University, Düsseldorf, Germany; t University of Chicago, Chicago, Illinois; u Baylor Scott & White Health, Advanced Heart Failure Program Clinic, Temple, Texas; v Inova Heart and Vascular Institute, Inova Fairfax Campus, Falls Church, Virginia; w Beth Israel Deaconess Medical Center, Boston, Massachusetts; x Center for Cardiovascular Analytics, Research, & Data Science (CARDS), Providence St. Joseph Research Network, Portland, Oregon; y Inova Heart and Vascular Institute, Inova Fairfax Campus, Falls Church, Virginia; z Division of Cardiovascular Medicine, Brigham and Women’s Hospital, Boston, Massachusetts; aa Cardiovascular Institute at Allegheny Health Network, Pittsburgh, Pennsylvania; bb University of Pittsburgh Medical Center, Pittsburgh, Pennsylvania; cc University of Oklahoma Health Science Center, Oklahoma City, Oklahoma; dd University of Nebraska Medical Center, Omaha, Nebraska; ee University of Chicago, Chicago, Illinois; ff Northwestern Medicine, Chicago, Illinois; gg Baylor University Medical Center, Dallas, Texas; hh University of Texas Medical Branch, Galveston, Texas; ii Maine Medical Center, Portland, Oregon; jj Houston Methodist Research Institute, Houston, Texas; kk Thomas Jefferson University Hospital, Philadelphia, Pennsylvania; ll Columbia University Irving Medical Center, New York, New York; mm UT Southwestern, Dallas, Texas; nn Atrium Health Sanger Heart and Vascular Institute, Charlotte, North Carolina; oo Banner University Medical Center, Phoenix, Arizona; pp Indiana University School of Medicine, Indianapolis, Indiana; qq Instituto Nacional de Cardiologia Ignacio Chavez, Mexico City, Mexico; rr Nippon Medical School, Tokyo, Japan; ss Oregon Health State University, Portland, Oregon; tt Tampa General Hospital, Tampa, Florida; uu TriStar Centennial Medical Center, Nashville, Tennessee; vv University of Pennsylvania, Philadelphia, Pennsylvania; ww University of Washington Medical Center, Seattle, Washington; xx Johns Hopkins University, Baltimore, Maryland; yy Atlantic Health System, Morristown, New Jersey; zz Baylor Scott and White, Plano, Texas; aaa Baylor Scott and White, Fort Worth, Texas; bbb Brown University Health, Providence, Rhode Island; ccc Houston Methodist Hospital, Houston, Texas; ddd Cardiovascular Research Foundation, New York, New York; a The Cardiovascular Center, Tufts Medical Center, Boston, Massachusetts; b Department of Cardiology, University Heart and Vascular Center Hamburg, University Medical Center Hamburg-Eppendorf, Hamburg, Germany; c German Center for Cardiovascular Research (DZHK), Partner Site Hamburg/Kiel/Lübeck, Hamburg, Germany; d Brown University Health Cardiovascular Institute, Providence, Rhode Island; e Beth Israel Deaconess Medical Center, Boston, Massachusetts; f Institute for Advanced Cardiac Care, Medical City Healthcare, Dallas, Texas; g Massachusetts General Hospital, Boston, Massachusetts; h Department of Cardiology, Pulmonology, and Vascular Medicine, Medical Faculty and University Hospital Düsseldorf, Heinrich-Heine-University, Düsseldorf, Germany; i University of Chicago, Chicago, Illinois; j Baylor Scott & White Health, Advanced Heart Failure Program Clinic, Temple, Texas; k Inova Heart and Vascular Institute, Inova Fairfax Campus, Falls Church, Virginia; l Center for Cardiovascular Analytics, Research, & Data Science (CARDS), Providence St. Joseph Research Network, Portland, Oregon; m Cardiovascular Research Foundation, New York, New York

**Keywords:** cardiogenic shock, intra-aortic balloon pump, outcome, transfer

## Abstract

**Background:**

The intra-aortic balloon pump (IABP) is a commonly used temporary mechanical circulatory support device in cardiogenic shock (CS). A substantial proportion of patients receive IABP at referring hospitals prior to transfer. The aim of this study was to compare clinical characteristics, treatment strategies, and outcomes between transferred and nontransferred IABP-treated patients with all-cause CS, acute myocardial infarction–related CS (AMI-CS), and heart failure–related CS (HF-CS).

**Methods:**

Intra-aortic balloon pump–treated CS patients from the multicenter Cardiogenic Shock Working Group registry (2020-2024) were analyzed. Adjusted logistic regression models were used to assess associations between transfer status and in-hospital mortality, native heart survival, heart replacement therapies, and in-hospital complications.

**Results:**

Among 2112 IABP-treated patients (36.6% AMI-CS, 48.9% HF-CS), 652 (30.9%) were primarily treated at referring centers and transferred. Transferred patients more frequently had AMI-CS (57.8% vs 27.1%) and less often HF-CS (27.5% vs 58.4%) than nontransferred subjects. Transfer was associated with higher in-hospital mortality (33.1% vs 26.4%; adjusted odds ratio [aOR], 1.38; 95% CI, 1.13-1.68; *P* < .001), with the strongest association observed in non–AMI-CS/non–HF-CS (40.6% vs 28.3%; aOR, 1.73; 95% CI, 1.07-3.04; *P* = .026). Complications were linked to transfer status, including stroke (aOR, 1.83; 95% CI, 1.30-2.57; *P* < .001), limb ischemia (aOR, 2.17; 95% CI, 1.53-3.09; *P* < .001), and in-hospital cardiac arrest (aOR, 1.37; 95% CI, 1.08-1.72; *P* = .006).

**Conclusions:**

Transfer status was independently associated with higher in-hospital mortality and complications. These findings emphasize the importance of structured referral pathways and heightened awareness at hub centers for this potentially high-risk IABP-treated CS cohort.

## Introduction

Cardiogenic shock (CS), as a life-threatening condition, is characterized by a significant decline in cardiac output, leading to critical end-organ hypoperfusion.^1^, ^2^, ^3^ Although major advances have been made in the understanding of epidemiology, etiology, severity staging, phenotyping, and treatment approaches, short-term mortality in CS remains high and often exceeds 40%.^2^^,^^4^, ^5^, ^6^, ^7^, ^8^, ^9^ Although early revascularization and temporary microaxial flow pump improve survival in selected acute myocardial infarction–related CS (AMI-CS) cohorts, evidence for effective pharmacologic or device-based therapies across the broader CS population is still lacking.^8^^,^^10^, ^11^, ^12^, ^13^, ^14^, ^15^, ^16^, ^17^, ^18^ This is particularly true for the heterogeneous subgroup of heart failure–related CS (HF-CS), which is managed with considerable variability across centers and health care systems worldwide.^8^^,^^10^^,^^14^ In the recent Altshock-2 trial, early intra-aortic balloon pump (IABP) support did not improve 60-day survival or successful bridging to heart replacement therapy (HRT) compared with standard care in acute decompensated HF-CS.^19^ Despite a lack of evidence supporting temporary mechanical circulatory support (tMCS) in HF-CS, the IABP remains the most used device in the US.^14^^,^^20^

Therapeutic decision-making in CS remains a clinical challenge that requires an interdisciplinary and standardized team-based approach.^5^^,^^21^^,^^22^ Advanced CS treatment approaches, including the availability of specialized cardiac care, tMCS devices, and HRT, are often lacking in low-volume CS centers. These centers depend on reliable communication and timely transfer to tertiary care institutions within a standardized hub-and-spoke framework.^22^ Observational studies have shown inconsistent mortality outcomes among transferred CS patients, with some suggesting a trend toward higher mortality.^23^^,^^24^ Interestingly, nearly half of CS patients at high-volume centers are transferred from other hospitals, with many already receiving IABP support externally prior to transfer.^23^ However, data on the clinical characteristics and outcomes of this tMCS-treated subgroup remain limited.

Hence, this study aimed to assess differences in clinical characteristics and outcomes between patients with externally implanted IABP and subsequent transfer versus those treated with IABP exclusively at tertiary care centers, using data from the large, multicenter Cardiogenic Shock Working Group (CSWG) registry.

## Methods

### Data source

This analysis was conducted using the international, multicenter CSWG registry, established by an academic research consortium, collecting data from across 36 participating centers with CS treatment expertise. Details on the definition of CS, eligibility criteria, and data entry procedures for the CSWG registry, predefined by principal investigators, have been previously published.^25^, ^26^, ^27^

In summary, the registry focuses on a standardized set of data elements, including patient demographics, medical history, laboratory values, hemodynamic parameters, and different treatment approaches (conservatively or with tMCS) at baseline and at predefined time points over the first 72 hours. Data collection is conducted through both prospective and retrospective arms. CS was adjudicated by the treating physicians at each participating center and defined as a sustained episode of cardiac dysfunction, fulfilling at least 1 of the following criteria: systolic blood pressure <90 mm Hg for a minimum of 30 minutes, requirement for vasoactive medications, cardiac index <2.2 L/min/m^2^ in the absence of hypovolemia, or initiation of 1 or more tMCS devices in the context of suspected CS. CS management and treatment approach was not predefined and was determined at the discretion of the local treating physicians at participating CSWG centers. To ensure data quality, case adjudication, centralized audits, and discrepancy resolution were performed by the CSWG principal investigators in close coordination with the contributing sites.

CS patients enrolled between 2020 and 2024 were included in the final analysis. CS was etiologically classified as AMI-CS (defined as a primary diagnosis of non–ST-segment elevation myocardial infarction or ST-segment elevation myocardial infarction [STEMI]), HF-CS (including de novo heart failure [HF] or acute-on-chronic HF), or non–AMI-CS/non–HF-CS (including arrhythmia induced, post-cardiotomy shock, graft dysfunction post–heart transplantation, or unknown CS etiology). Society for Cardiovascular Angiography & Interventions (SCAI) stages were assigned based on the CSWG-SCAI definition, as previously published.^26^ Out-of-hospital cardiac arrest was not used as a standalone criterion for classification as SCAI stage E. In transferred patients, CSWG-SCAI classification was assigned upon arrival at the hub center (as a reliable SCAI assessment during the referring-center stay was not available), whereas in nontransferred patients, it was determined from the beginning at the time of initial presentation at the CSWG site.

This analysis was conducted in accordance with the Declaration of Helsinki and received approval for patient enrollment from the institutional review boards or local ethics committees at each participating center.

### Definition of study groups

CS patients treated with IABP as the initial tMCS device were included in this analysis. Patients were subsequently categorized into 2 groups based on the location of IABP initiation. The transfer group consisted of patients who were transferred to CSWG sites and received IABP implantation at the transferring center, whereas the nontransfer group included those directly admitted to CSWG sites, where they subsequently underwent IABP implantation.

For transferred IABP-treated patients, data were collected and reported both at the time of index hospitalization at the transferring (spoke) center and upon arrival at the receiving CSWG (hub) site. Among the transfer and nontransfer groups, all patients were analyzed as an overall cohort (all-cause CS) and stratified into AMI-CS, HF-CS, and non–AMI-CS/non–HF-CS.

### Outcome

The primary outcome of this study was in-hospital mortality. Secondary outcomes included in-hospital HRT, defined as left ventricular assist device (LVAD) implantation or heart transplantation during the index hospitalization, and native heart survival. Native heart survival was defined as discharge from the index hospitalization without undergoing HRT. Safety end points included in-hospital complications such as stroke, in-hospital cardiac arrest, limb ischemia, acute kidney injury, bleeding requiring surgery, bleeding requiring transfusion, and hemolysis.

### Statistical analyses

Binary variables are presented as absolute numbers and relative frequencies, and comparisons were conducted using Pearson’s χ^2^ test or Fisher exact test. Continuous variables are shown as the median with interquartile range (IQR) and analyzed using the Kruskal-Wallis test.

In-hospital mortality was assessed in all patients, without missing data for this outcome. To evaluate the association between transfer status (independent variable) and mortality, HRT, native heart survival, or distinct in-hospital complications (dependent variables), univariable and multivariable logistic regression models (adjusted for age and sex) were fitted in the overall cohort and in prespecified subgroups.

To assess the association between transfer status (independent variable) and the probability of receiving a second tMCS device, vasoactive drug use, or mechanical ventilation (dependent variables), logistic regression models were performed.

Odds ratios and 95% CI are presented; a *P* value below .05 was considered statistically significant. Analyses were performed using R statistical software (version 4.4.1).

## Results

### Study cohort

Among 11,244 patients in the CSWG registry, 2112 patients treated with IABP met the inclusion criteria for this study (Central Illustration). The median age was 63 years (IQR, 54-70), 623 (29.5%) were women, and 1032 (48.9%) presented with HF-CS, and 772 (36.6%) with AMI-CS. Baseline lactate was 2.2 mmol/L (IQR, 1.5-3.7), creatinine was 1.4 mg/dL (IQR, 1.1-2.0), the left ventricular ejection fraction was 22.5% (IQR, 15-35), and a total of 229 (10.9%) IABP-treated patients had a prior out-of-hospital cardiac arrest.

**Central Illustration:**
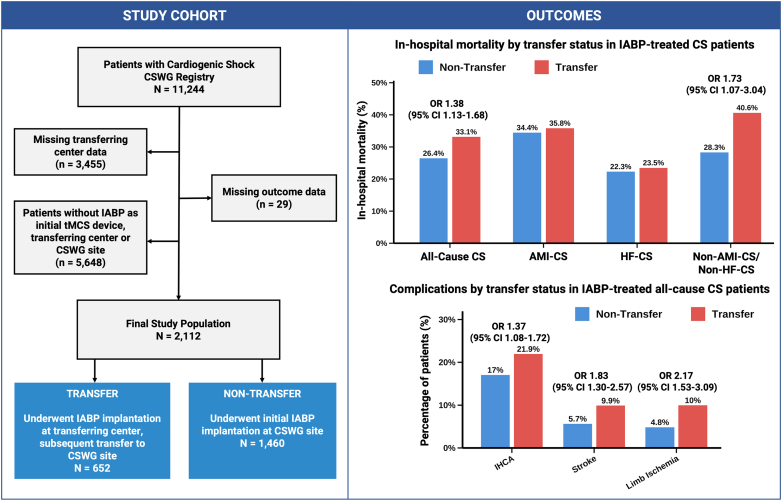
**Outcomes in transferred intra-aortic balloon pump (IABP)-supported cardiogenic shock (CS) patients.** AMI-CS, acute myocardial infarction–related cardiogenic shock; CSWG, Cardiogenic Shock Working Group; HF-CS, heart failure–related cardiogenic shock; IHCA, in-hospital cardiac arrest; OR, odds ratio; tMCS, temporary mechanical circulatory support.

**Figure 1:**
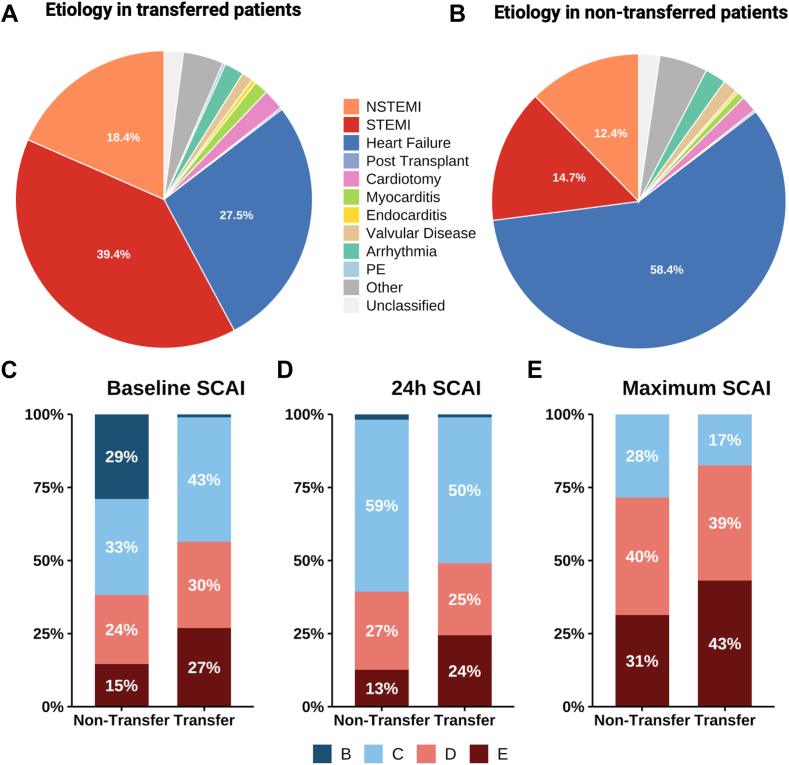
**Etiology and Society for Cardiovascular Angiography & Interventions (SCAI) staging in transferred vs nontransferred intra-aortic balloon pump (IABP)-treated patients.** (**A**) Distribution of etiology in transferred IABP-treated patients. (**B**) Distribution of etiology in nontransferred IABP-treated patients. Cardiogenic Shock Working Group (CSWG)-SCAI shock stages at baseline (**C**), 24 hours (**D**), and maximum during hospitalization (**E**) in transferred versus nontransferred IABP-treated patients. CSWG-SCAI staging for transferred patients was assessed upon arrival at the CSWG site, as reliable SCAI assessment during the referring-center stay was not available. In this context, baseline CSWG-SCAI stage B assignment was possible only in those cases in which the IABP had been explanted prior to transfer (1%). NSTEMI, non–ST-segment elevation myocardial infarction; PE, pulmonary embolism; STEMI, ST-segment elevation myocardial infarction.

Of the total IABP cohort, 652 patients (30.9%) received IABP support at the transferring institution and were subsequently transferred, whereas 1460 patients (69.1%) were initially supported with IABP at CSWG sites. Baseline characteristics of the overall cohort and stratified by transfer status are presented in Tables 1 and 2. Transferred patients were less frequently female (26.5% vs 30.9%, *P* = .044) and presented with a lower overall comorbidity burden, including lower rates of atrial fibrillation (19.4% vs 34.2%, *P* < .001), chronic kidney disease (18.9% vs 35.9%, *P* < .001), and history of stroke/transient ischemic attack (9.7% vs 14.6, *P* = .002), compared to patients who presented directly to and underwent IABP implantation at a CSWG site. A known history of HF was also significantly less frequently present among patients transferred with an IABP (39.8% vs 68.3%, *P* < .001). The distribution of etiology in transferred and nontransferred IABP-supported patients is illustrated in Figure 1A and B. Transferred patients were more frequently treated with IABP for AMI-CS (57.8% vs 27.1%, *P* < .001), with a high proportion of STEMI (39.4% vs 14.7%), and less often for HF-CS (27.5% vs 58.4%, *P* < .001), compared to nontransferred patients. Out-of-hospital cardiac arrest was more prevalent in the transfer group (20.5% vs 6.6%, *P* < .001) compared to the nontransfer group. Blood pressure profiles and invasive hemodynamic parameters did not differ significantly across groups. Transferred patients were more likely to present with higher baseline, higher 24-hour, and higher maximum SCAI shock stages during hospitalization (assessed at the CSWG site), compared to nontransferred patients (Figure 1C-E). Characteristics of the AMI-CS and HF-CS cohorts are detailed in Supplemental Tables S1-S4.

**Table 1:** Characteristics of IABP-treated patients stratified by transfer status

Characteristic	Overall (N = 2112)	Nontransferred IABP-treated patients (n = 1460)	Transferred IABP-treated patients (n = 652)	*P* value
Female sex	29.5 (623/2110)	30.9 (450/1458)	26.5 (173/652)	.044
Age, y	63 (54-70)	63 (53-69)	63 (55-70)	.077
Body mass index, kg/m^2^	27.9 (24.1-32.1)	27.6 (23.8-31.9)	28.5 (24.7-32.6)	.011
Medical history				
Hypertension	67.3 (1417/2105)	67.8 (986/1454)	66.2 (431/651)	.47
Diabetes mellitus	43.6 (918/2104)	43.7 (635/1454)	43.5 (283/650)	.95
Atrial fibrillation/flutter	29.6 (624/2105)	34.2 (498/1455)	19.4 (126/650)	<.001
Chronic kidney disease	30.6 (645/2107)	35.9 (522/1456)	18.9 (123/651)	<.001
Peripheral vascular disease	8.7 (183/2104)	9.3 (135/1454)	7.4 (48/650)	.15
COPD	11.6 (244/2103)	12.8 (186/1453)	8.9 (58/650)	.010
Asthma	7.8 (158/2028)	8.6 (121/1408)	6.0 (37/620)	.042
Cancer	11.1 (229/2067)	12.4 (177/1432)	8.2 (52/635)	.005
Liver disease	3.7 (77/2061)	4.5 (65/1430)	1.9 (12/631)	.004
Anemia	18.9 (386/2047)	21.2 (301/1423)	13.6 (85/624)	<.001
History of stroke/TIA	13.1 (275/2107)	14.6 (212/1456)	9.7 (63/651)	.002
Severe valve disease	21.4 (450/2107)	25.0 (364/1456)	13.2 (86/651)	<.001
Prior CAD	46.3 (937/2022)	46.1 (647/1403)	46.8 (290/619)	.76
History of HF	59.5 (1252/2104)	68.3 (994/1456)	39.8 (258/648)	<.001
History of MI	24.0 (506/2105)	24.7 (360/1456)	22.5 (146/649)	.27
No. of comorbidities	4 (2, 6)	4.0 (2, 6)	3.0 (1, 5)	<.001
Etiology of shock				
AMI-CS	36.6 (772/2112)	27.1 (395/1460)	57.8 (377/652)	<.001
HF-CS	48.9 (1032/2112)	58.4 (853/1460)	27.5 (179/652)	<.001
Non–AMI-CS/non–HF-CS	14.6 (308/2112)	14.5 (212/1460)	14.7 (96/652)	.89
OHCA	10.9 (229/2092)	6.6 (96/1444)	20.5 (133/648)	<.001
LVEF baseline hub center	22.5 (15.0, 35.0)	22.5 (15.0, 35.0)	22.5 (17.0, 35.0)	.051
SCAI shock stage				
SCAI B baseline	17.6 (237/1347)	28.9 (231/799)	1.1 (6/548)	<.001
SCAI C baseline	36.8 (496/1347)	32.9 (263/799)	42.5 (233/548)	<.001
SCAI D baseline	26.1 (351/1347)	23.7 (189/799)	29.6 (162/548)	.015
SCAI E baseline	19.5 (263/1347)	14.5 (116/799)	26.8 (147/548)	<.001
SCAI B 24 h	1.5 (15/986)	1.8 (12/670)	0.9 (3/316)	.41
SCAI C 24 h	56.0 (552/986)	58.8 (394/670)	50.0 (158/316)	.009
SCAI D 24 h	26.2 (258/986)	26.9 (180/670.0)	24.7 (78/316)	.47
SCAI E 24 h	16.3 (161/986)	12.5 (84/670)	24.4 (77/316)	<.001
SCAI B Max	0.0 (0/394)	0.0 (0/211)	0.0 (0/183)	—
SCAI C Max	23.4 (92/394)	28.4 (60/211)	17.5 (32/183)	.010
SCAI D Max	39.8 (157/394)	40.3 (85/211)	39.3 (72/183)	.85
SCAI E Max	36.8 (145/394)	31.3 (66/211)	43.2 (79/183)	.015
IABP + second MCS device				
VA-ECMO	14.0 (296/2112)	12.1 (177/1460)	18.3 (119/652)	<.001
Impella CP	5.6 (119/2112)	3.9 (57/1460)	9.5 (62/652)	<.001
Impella 5.0	0.4 (9/2112)	0.5 (8/1460)	0.2 (1/652)	.29
Impella 5.5	16.4 (347/2112)	16.6 (243/1460)	16.0 (104/652)	.69
IABP access site				<.001
Axillary	16.7 (257/1540)	15.7 (227/1448)	32.6 (30/92)	
Femoral	83.3 (1283/1540)	84.3 (1221/1448)	67.4 (62/92)	
No. of MCS devices (during stay)				<.001
2	88.6 (1872/2112)	86.0 (1255/1460)	94.6 (617/652)	
≥3	11.4 (240/2112)	14.0 (205/1460)	5.4 (35/652)	
Other treatments				
No. of vasoactive drugs (during stay)				<.001
0	4.3 (54/1248)	3.4 (30/894)	6.8 (24/354)	
1	28.2 (352/1248)	29.8 (266/894)	24.3 (86/354)	
2	29.1 (363/1248)	31.1 (278/894)	24.0 (85/354)	
3	23.0 (287/1248)	21.1 (189/894)	27.7 (98/354)	
≥4	15.4 (192/1248)	14.7 (131/894)	17.2 (61/354)	
Mechanical ventilation	77.0 (1616/2098)	76.2 (1105/1450)	78.9 (511/648)	.18
Renal replacement therapy	23.7 (500/2106)	23.8 (346/1454)	23.6 (154/652)	>.93

Binary variables are presented as percentage (n/N), and comparisons were conducted using Pearson’s χ^2^ test or Fisher exact test. Continuous variables are shown as the median (IQR) and were compared using the Wilcoxon rank-sum test.

AMI-CS, acute myocardial infarction–related cardiogenic shock; CAD, coronary artery disease; COPD, chronic obstructive pulmonary disease; HF, heart failure; HF-CS, heart failure–related cardiogenic shock; IABP, intra-aortic balloon pump; LVEF, left ventricular ejection fraction; MCS, mechanical circulatory support; MI, myocardial infarction; OHCA, out-of-hospital cardiac arrest; SCAI, Society for Cardiovascular Angiography & Interventions; VA-ECMO, veno-arterial extracorporeal membrane oxygenation; TIA, transient ischemic attack.

**Table 2:** Laboratory and hemodynamic characteristics of IABP-treated patients stratified by transfer status

Characteristic	Overall	Transferred IABP-treated patients			Nontransferred IABP-treated patients	*P* value (overall)	*P* value (pairwise)^a^
		On admission at transferring center	Before transfer	On arrival at the CSWG site	Admission at the CSWG site		
Lactate, mEq/L	2.2 (1.5-3.7)	3.3 (1.9-6.6); n = 163	2.3 (1.5-4.5); n = 226	1.7 (1.2-3.4); n = 576	2.0 (1.4-3.3); n = 968	<.001	<.001
pH	7.4 (7.3-7.4)	7.3 (7.2-7.4); n = 122	7.4 (7.3-7.4); n = 196	7.4 (7.3-7.4); n = 434	7.4 (7.3-7.4); n = 579	<.001	<.001
ALT, IU/L	36 (19-91)	45 (22-114); n = 289	81 (32-358); n = 247	73 (32-264); n = 596	34 (19-87); n = 1238	<.001	.005
AST, IU/L	43 (25-119)	56 (26-187); n = 285	134 (45-409); n = 245	138 (43-442); n = 597	41 (24-105); n = 1253	<.001	.001
Serum creatinine, mg/dL	1.4 (1.1-2.0)	1.38 (1.09-1.81); n = 354	1.40 (1.10-2.13); n = 338	1.38 (1.00-2.01); n = 647	1.41 (1.07-2.00); n = 1437	.26	.16
Systolic blood pressure, mm Hg	109 (97-125)	114 (99-133); n = 351	110 (98-126); n = 377	112 (97-129); n = 608	108 (97-124); n = 1440	<.001	<.001
Diastolic blood pressure, mm Hg	72 (62-82)	74 (61-87); n = 350	66 (55-75); n = 376	64 (53-79); n = 608	71 (63-81); n = 1439	<.001	.032
Heart rate, bpm	93 (78-108)	95 (79-111); n = 348	90 (75-104); n = 377	88 (75-102); n = 648	92 (78-107); n = 1444	<.001	.031
MAP, mm Hg	83 (74-95)	88 (71-103); n = 207	82 (70-92); n = 264	82 (72-95); n = 582	83 (74-93); n = 1101	.021	.032
RAP, mm Hg	14 (10-19)	14 (10-19); n = 152	13 (9-18); n = 126	13 (9-17); n = 287	13 (9-18); n = 100	.11	.071
Pulmonary artery systolic pressure, mm Hg	45 (37-56)	46 (37-55); n = 162	—	43 (33-52); n = 319	45 (38-59); n = 104	.002	>.92
Pulmonary artery diastolic pressure, mm Hg	25 (20-32)	24 (20-32); n = 161	22 (17-30); n = 148	23 (18-28); n = 316	26 (20-32); n = 97	.002	.48
Pulmonary capillary wedge pressure, mm Hg	25 (20-32)	25 (20-33); n = 138	25 (18-32); n = 76	20 (16-29); n = 82	24 (20-31); n = 45	.024	.82
Cardiac output, L/min	3.6 (2.7-4.6)	3.6 (2.8-4.6); n = 133	3.8 (3.0-4.9); n = 106	4.1 (3.2-5.5); n = 177	3.5 (2.7-4.6); n = 69	.002	>.94

Data are presented as median (IQR) and available n. Group comparisons were performed using the Kruskal-Wallis test.

ALT, alanine transaminase; AST, aspartate transaminase; IABP, intra-aortic balloon pump; MAP, mean arterial pressure; RAP, right atrial pressure.

a Pairwise comparison between admission at the transferring center (transfer) and admission at the CSWG site (nontransfer) was performed using the Wilcoxon rank-sum test.

### Transfer status associated with outcome

**Figure 2:**
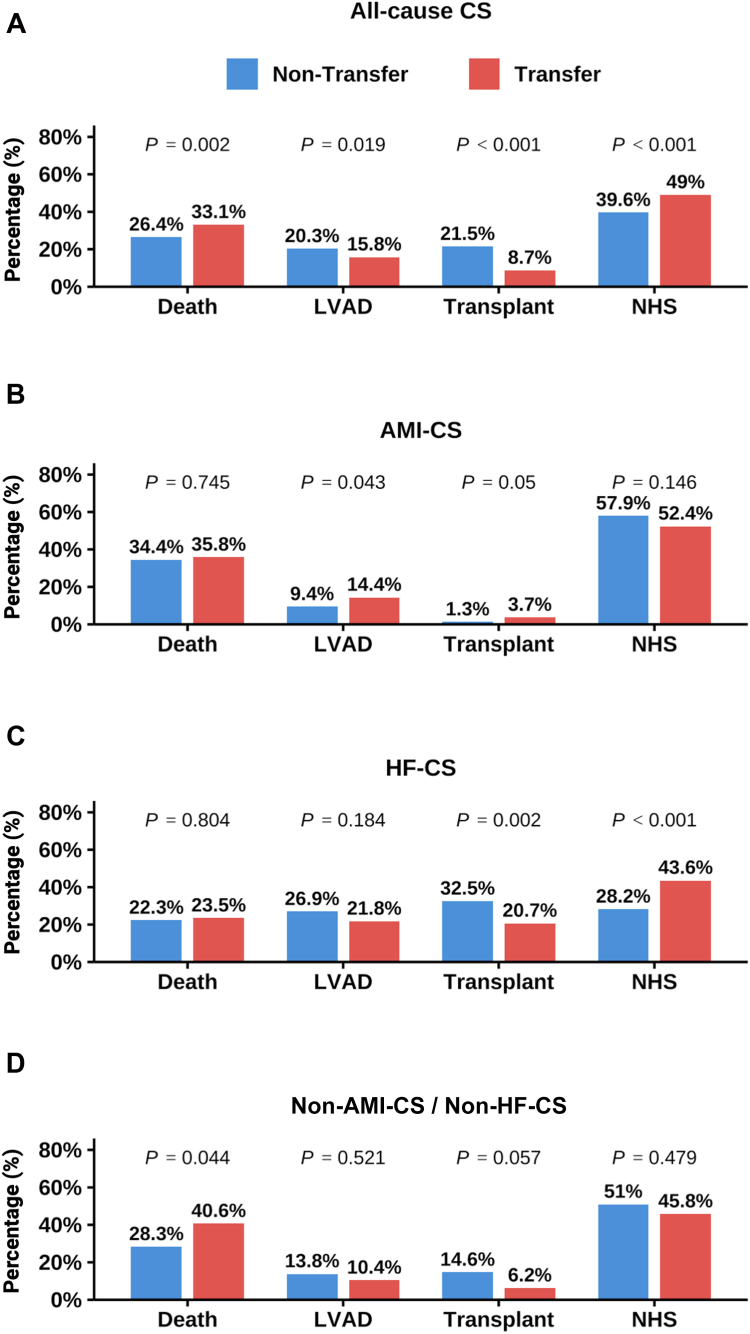
**Clinical outcome across different cardiogenic shock (CS) subtypes.** Crude clinical outcomes, including death (in-hospital mortality), heart replacement therapy with left ventricular assist device (LVAD) or heart transplantation, and native heart survival (NHS), in IABP-treated patients stratified by transfer status. Shown are outcomes for all-cause CS (**A**), acute myocardial infarction–related CS (AMI-CS) (**B**), heart failure–related CS (HF-CS) (**C**), and non–AMI-CS/non–HF-CS (**D**).

**Figure 3:**
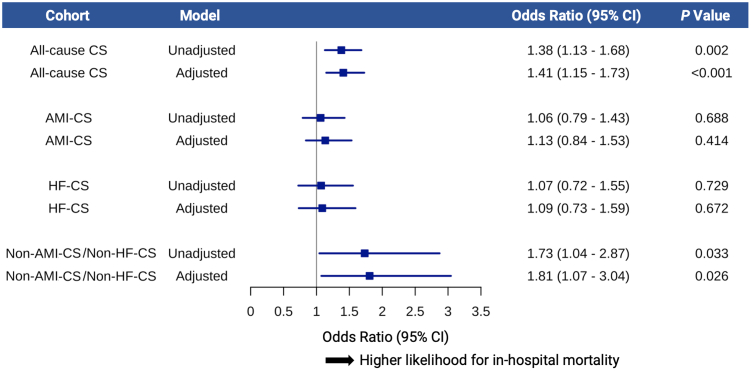
**Association between transfer status and in-hospital mortality.** Association between transfer status (transfer vs nontransfer) and in-hospital mortality across cardiogenic shock (CS) subtypes. Odds ratios for in-hospital mortality were calculated using multivariable logistic regression models in all-cause CS, acute myocardial infarction–related CS (AMI-CS), heart failure–related CS (HF-CS), and non–AMI-CS/non–HF-CS, adjusted for age and sex.

In the final study cohort, 602 (28.5%) IABP-treated patients died in-hospital. Among patients with all-cause CS, the crude in-hospital mortality was higher in transferred patients compared to nontransferred patients (33.1% vs 26.4%, *P* = .002; Figure 2). The corresponding adjusted OR [aOR] for transfer status was 1.41 (95% CI, 1.15-1.73; *P* < .001; Figure 3).

In subgroup analyses stratified by CS etiology, in-hospital mortality was similar between groups in AMI-CS (transfer vs nontransfer: 35.8% vs 34.4%; aOR, 1.13; 95% CI, 0.84-1.53; *P* = .414) and HF-CS (transfer vs nontransfer: 23.5% vs 22.3%; aOR, 1.09; 95% CI, 0.73-1.59; *P* = .672). Among patients with non–AMI-CS/non–HF-CS, in-hospital mortality was higher in the transfer group (transfer vs nontransfer: 40.6% vs 28.3%) and independently associated with 81% higher relative odds of in-hospital mortality (odds ratio, 1.81; 95% CI, 1.07-3.04; *P* = .026, Figure 3).

**Figure 4:**
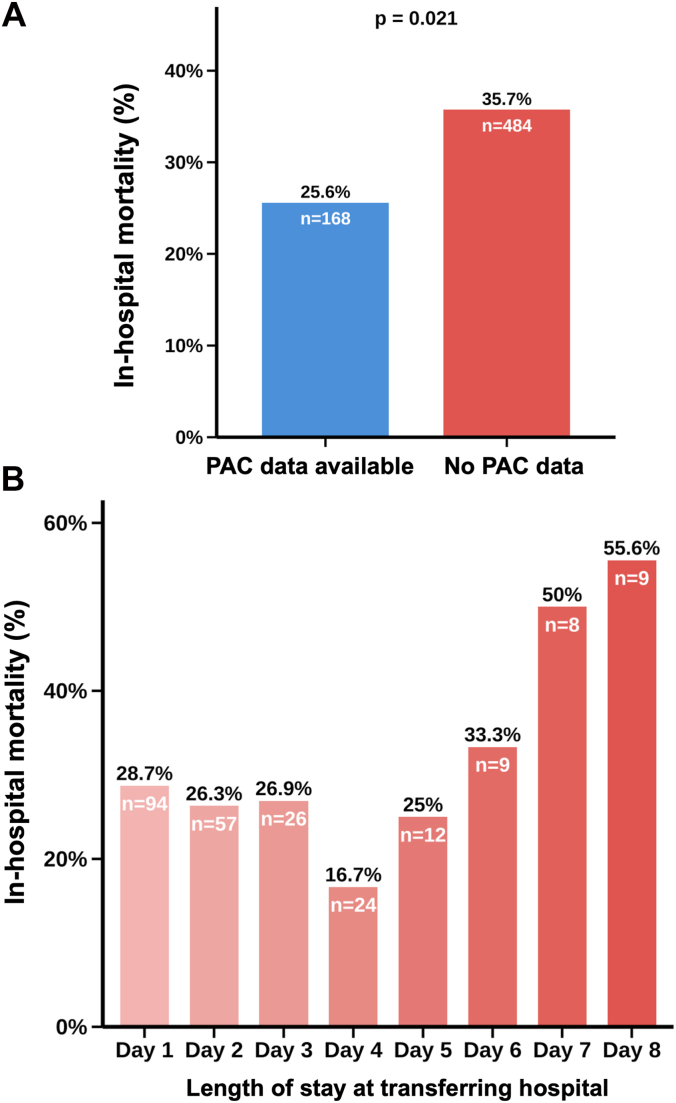
**In-hospital mortality by hemodynamic data availability and length of stay at the referring hospital.** (**A**) In-hospital mortality among transferred patients stratified by availability of pulmonary artery catheter (PAC) hemodynamic data at the referring hospital. (**B**) In-hospital mortality stratified by length of stay at the referring hospital (days 1-8) among transferred patients, representing the time from admission at the transferring hospital to admission at the hub center.

In transferred patients, in-hospital mortality was lower when pulmonary artery catheter hemodynamic data were available compared with when no pulmonary artery catheter data were recorded (25.6% vs 35.7%; *P* = .021; Figure 4A). In addition, in-hospital mortality increased over the first 8 days with a longer length of stay at the referring hospital prior to transfer (Figure 4B).

Transferred IABP-supported patients received less HRT compared to nontransferred patients with all-cause CS (transplantation: 8.7% vs 21.5%, *P* < .001; LVAD: 15.8% vs 20.3%, *P* = .019; Figure 2A). Specifically, transfer status was independently associated with lower odds of transplantation (aOR, 0.33; 95% CI, 0.24-0.45; *P* < .001; Supplemental Figure S1) and LVAD implantation (aOR, 0.72; 95% CI, 0.56-0.92; *P* = .011; Supplemental Figure S2) in all-cause CS, compared to nontransferred IABP-treated patients. Transfer status was also associated with a lower likelihood of transplant in HF-CS (aOR, 0.53; 95% CI, 0.35-0.78; *P* = .002) and non–AMI-CS/non–HF-CS (aOR, 0.35; 95% CI, 0.12-0.83; *P* = .027). In contrast, transferred IABP-treated patients with AMI-CS more frequently underwent transplantation than nontransferred patients (3.7% vs 1.3%, *P* = .050). The corresponding aOR was 2.88 (95% CI, 1.08-9.07; *P* = .047).

Native heart survival was more frequently observed among transferred IABP-treated patients with all-cause CS (49.0% vs 39.6%, *P* < .001) compared to nontransferred patients, mainly driven by transferred HF-CS patients (43.6% vs 28.2%, *P* < .001). In contrast, among patients with AMI-CS and non–AMI-CS/non–HF-CS, native heart survival was observed at lower rates in those who were transferred (Figure 2B-D).

### Transfer status associated with safety end points

**Figure 5:**
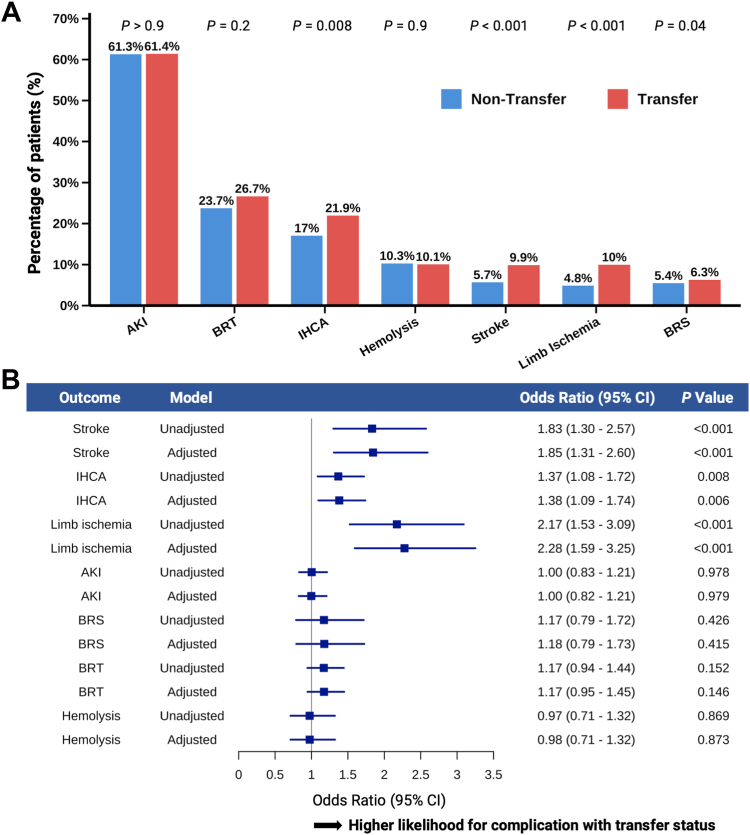
**Association between transfer status and in-hospital complications.** (**A**) Crude incidence of in-hospital complications in intra-aortic balloon pump–treated patients stratified by transfer status in all-cause cardiogenic shock. (**B**) Odds ratios for complications associated with transfer status (transfer vs nontransfer), using logistic regression models, adjusted for age and sex, in all-cause cardiogenic shock. AKI, acute kidney injury; BRS, bleeding requiring surgery; BRT, bleeding requiring transfusion; IHCA, in-hospital cardiac arrest.

Transferred IABP-treated patients experienced higher rates of in-hospital complications compared to nontransferred patients (Figure 5A). Specifically, in-hospital cardiac arrest (21.9% vs 17.0%, *P* = .008), stroke (9.9% vs 5.7%, *P* < .001), and limb ischemia (10.0% vs 4.8%, *P* < .001) occurred more frequently in transferred IABP-treated patients with all-cause CS. In the adjusted models, transfer status remained independently associated with an increased risk of complications for in-hospital cardiac arrest (aOR, 1.38; 95% CI, 1.09-1.74; *P* = .006), for stroke (aOR, 1.85; 95% CI, 1.31-2.50; *P* < .001), and for limb ischemia (aOR, 2.28; 95% CI, 1.59-3.25; *P* < .001) (Figure 5B). There were no significant differences in the odds of acute kidney injury, bleeding events, or hemolysis between transferred and nontransferred IABP-treated patients in logistic regression models. Subgroup analyses stratified by AMI-CS, HF-CS, and non–AMI-CS/non–HF-CS are provided in Supplemental Figures S3-S6.

### Transfer status and selected treatments

Among IABP-treated patients, Impella 5.5 was more frequently used as a second tMCS device during the hospital stay (16.4%) compared with VA-ECMO (14.0%) and Impella CP (5.6%). When stratified by transfer status, both VA-ECMO (18.3% vs 12.1%, *P* < .001) and Impella CP (9.5% vs 3.9%, *P* < .001) were more often used in transferred than in nontransferred patients, whereas the use of Impella 5.5 did not differ significantly between groups (16.0% vs 16.6%, *P* = .70).

Transferred patients were more likely to require multiple vasoactive drugs (aOR for all-cause CS, 1.47; 95% CI, 1.14-1.89; *P* = .003; Supplemental Figure S7). The rate of mechanical ventilation was high in both groups (78.9% in transferred vs 76.2% in nontransferred patients). In the adjusted models, transfer status was associated with an 84% higher likelihood of receiving mechanical ventilation (aOR, 1.84; 95% CI, 1.33-2.57; *P* < .001) in AMI-CS, without differences observed in other subgroups (Supplemental Figure S8). Transfer status was associated with prolonged ventilation duration both in all-cause CS (aOR, 1.52; 95% CI, 1.23-1.89; *P* < .001) and in AMI-CS (aOR, 1.43, 95% CI, 1.01-2.02; *P* = .042), but not in HF-CS (Supplemental Figure S9). The rate of renal replacement therapy (RRT) was similar between transferred and nontransferred patients (23.6% vs 23.8%). In subgroup analyses, transferred patients with HF-CS had a 40% lower likelihood of receiving RRT compared to nontransferred patients (aOR, 0.60; 95% CI, 0.38-0.91; *P* = .020). In contrast, among patients with non–AMI-CS/non–HF-CS, transferred patients had a higher likelihood of receiving RRT (aOR, 1.73; 95% CI, 1.04-2.88; *P* = .035; Supplemental Figure S10).

## Discussion

In this large, contemporary cohort of 2112 IABP-treated CS patients, nearly one-third underwent IABP implant at a transferring institution and were subsequently transferred to a higher level of care CSWG site. Among transferred patients, AMI-CS was the predominant etiology, with a high proportion of patients presenting with STEMI. Transfer status was associated with higher in-hospital mortality compared with nontransferred IABP-treated patients, with the strongest association observed in the non–AMI-CS/non–HF-CS subgroup. In transferred patients, lower mortality was observed with early pulmonary artery catheter use and when the pretransfer hospital stay was shorter. Transfer status was linked to a higher burden of in-hospital complications, including higher rates of stroke, limb ischemia, and in-hospital cardiac arrest.

### Transfer status associated with outcome

Although nearly half of the patients with CS treated at high-volume centers are transferred from another hospital, data comparing clinical characteristics, treatment strategies, and outcomes between transferred and nontransferred patients remain limited.^23^ Among transferred patients, IABP remains the most frequently used initial tMCS device and continues to be the most widely used tMCS modality in the US.^14^^,^^20^^,^^23^ Prior randomized trials have failed to demonstrate a survival benefit of IABP use in AMI-CS. The Intra-aortic Balloon Pump in Cardiogenic Shock II trial showed no benefit in 30-day, 1-year, or 6-year mortality in CS complicating AMI undergoing early revascularization.^15^^,^^28^^,^^29^ In the recent ALTSHOCK-2 trial, early IABP plus standard of care did not improve 60-day survival or successful bridging to HRT compared with standard of care alone in acute decompensated HF-CS.^19^ The limited generalizability of existing tMCS trials, often restricted to highly selected patient cohorts, likely contributes to continued uncertainty among treating physicians regarding its optimal use. Data on clinical characteristics and outcomes of CS patients initially treated with IABP at referring centers and subsequently transferred to tertiary care remain limited, despite representing a sizable subgroup. In this study, 30.9% of IABP-treated patients were transferred from referring centers to tertiary care. Interestingly, 39.4% of transferred IABP-treated patients had STEMI as the most likely underlying CS etiology, compared to only 14.7% among nontransferred patients. These high STEMI proportions may reflect practice patterns driven by the limited availability of alternative tMCS devices at referring centers, where IABP remains commonly used because of its relative ease of implantation with a smaller sheath size compared to other tMCS devices. As these data were collected in the pre-DanGer Shock era (2020-2024), the impact of emerging evidence on future practice patterns remains to be seen.^13^

In this study, transfer status was associated with higher in-hospital mortality compared with nontransferred IABP-treated patients. These findings align with prior observational data reporting higher mortality among transferred CS patients (37% vs 29%) compared with those initially managed at tertiary centers.^23^ Several factors may explain these findings. First, referring centers, often lower-volume hospitals, may have limited access to advanced CS therapies and reduced capability for early CS assessment, escalation of care, intensive care resources, and upstream tMCS implementation.^22^^,^^30^ When advanced care exceeds local resources, current guidelines now recommend transfer to CS centers with established infrastructure for advanced CS management, enhancing outcome.^31^

Second, transfer-related delays themselves in therapeutic escalation may worsen outcomes, including a delayed second-device tMCS implantation and an increased need for vasoactive support to maintain perfusion during ongoing hemodynamic deterioration. The timing of tMCS initiation may critically affect efficacy, yet the optimal window for tMCS implementation and its use as upstream therapy remains unclear.^32^^,^^33^ In this study, the exact timing of device implantation or escalation at the referring hospital was not available. However, in-hospital mortality increased with longer length of stay at the referring center prior to transfer.

Third, transferred patients more frequently presented with worse CS severity at hub center arrival, with higher rates of SCAI D/E CS compared to nontransferred patients (57% vs 39%), suggesting that clinical deterioration may have prompted interhospital transfer. Transferred patients continued to show higher shock severity at 24 hours at the hub center (D/E: 49% vs 40%) and ultimately reached higher maximum SCAI stages during their hospital course (D/E: 82% vs 71%). These trends may also reflect delayed escalation to a second tMCS device or delayed recognition of worsening hemodynamics at the referring center. In this study, early invasive hemodynamic monitoring was linked to improved outcomes, as in-hospital mortality was approximately 10% lower in transferred patients for whom early pulmonary artery catheter-derived hemodynamic data were available compared with those without pulmonary artery catheter measurements. Notably, such delays may also contribute to the higher associated need for multiple vasoactive agents among transferred compared to nontransferred IABP-treated patients.

Finally, optimal management of CS requires a coordinated, multidisciplinary team-based approach, an infrastructure often lacking at low-volume centers. As a team-based approach to CS management has been associated with improved clinical outcomes, the absence of such structures at referring centers may further compound the risk of adverse outcomes in transferred patients.^5^^,^^21^^,^^22^

In subgroup analyses stratified by CS etiology, transfer status was not associated with in-hospital mortality in AMI-CS or HF-CS. However, among patients with non–AMI-CS/non–HF-CS, transfer was linked to significantly higher in-hospital mortality. A multicenter observational study has similarly found no mortality difference in transferred AMI-CS patients.^23^ This may reflect the presence of standardized treatment algorithms for AMI-CS, including guideline-recommended immediate reperfusion strategies and streamlined transfer protocols. AMI-CS is often promptly recognized because of its abrupt onset with hypotension and early signs of hypoperfusion, resulting in a rapid clinical trajectory that facilitates timely diagnosis and management.^34^ In contrast, patients with HF-CS often present with progressive congestion and delayed hypotension, resulting in a slower and more variable clinical course, which may complicate early recognition and triage, particularly at nonspecialized CS centers. However, improved awareness, updated practical recommendations, and structured referral pathways for advanced HF therapies may help explain the comparable outcomes observed in transferred and nontransferred HF-CS patients.^18^

Moreover, transferred IABP-treated patients are likely subject to referral bias, as referring centers may primarily transfer the most rapidly deteriorating patients, particularly those still considered to have a chance of recovery. In this study, transferred HF-CS patients demonstrated higher native heart survival rates and a lower comorbidity burden. As higher comorbidity burden is associated with worse clinical outcomes, especially in the HF-CS subset, this pattern may reflect preselection for advanced HRT such as LVAD or transplant, potentially biasing outcomes toward more favorable profiles in this transferred subset.^35^

The most pronounced mortality difference was observed in patients with non–AMI-CS/non–HF-CS. Several factors may contribute to this finding. First, these patients often present with uncommon or less clearly defined etiologies (eg, myocarditis, tachyarrhythmias, post–cardiotomy shock, or pulmonary embolism), lacking standardized care pathways. Second, the initial presentation is frequently ambiguous, leading to delayed diagnosis or misclassification at the referring center. Third, specialized diagnostics and disease-specific expertise are often required but not immediately available, which could further lead to transfer delays. Finally, the lack of guideline-directed CS strategies leaves structured escalation pathways for non–AMI-CS/non–HF-CS poorly defined, and the clinical role of tMCS in this subgroup remains uncertain.

### Transfer status associated with in-hospital complications

The use of tMCS is inherently associated with an increased risk of complications, largely driven by the requirement for large-bore vascular access and its effects on hemostasis and the coagulation system.^10^^,^^36^, ^37^, ^38^ Although IABP requires smaller sheath sizes compared to other tMCS devices, it remains associated with procedural risks, bleeding, and downstream complications over the course of support. Comparative data on complication rates between transferred and nontransferred IABP-treated patients remain scarce. In this study, transferred IABP-treated patients exhibited higher rates of in-hospital complications, including bleeding requiring transfusion, in-hospital cardiac arrest, stroke, and limb ischemia. These increased risks may be amplified in the transfer setting, where factors such as prolonged support duration, more advanced shock severity, or limited institutional experience with tMCS may adversely affect the overall risk-benefit profile. Further research is needed to better characterize complication risk and to inform management strategies in transferred tMCS-treated patients.

### Limitations

This study has several limitations. First, it is based on a nonrandomized dataset, which precludes causal inference. Second, only short-term and in-hospital outcomes were available, limiting conclusions about long-term effects. Third, transfer decisions were not protocolized and likely influenced by local clinical judgment, resource availability, and institutional pathways. This introduces potential selection bias, particularly regarding which patients were considered suitable for transfer while on IABP support. Treatment decisions regarding vasoactive drugs and tMCS were made at the discretion of the treating teams and likely influenced by provider preference and institutional practice patterns. Granular data on transfer logistics, such as timing of clinical deterioration, transport delays, and pretransfer stabilization, were not systematically collected, limiting insights into interhospital transfer dynamics and their potential impact on outcomes. Finally, comparison with patients who received an IABP at the referring center but were not transferred was not possible, as these data are not captured within the current registry. Future studies, particularly those focused on transfer processes, should aim to include this patient group to better characterize selection patterns and their impact on outcomes.

## Conclusion

In this large, multicenter, real-world cohort of 2112 IABP-treated CS patients, interhospital transfer was associated with higher CS severity, increased in-hospital mortality, and higher rates of complications, including stroke, limb ischemia, and cardiac arrest. These findings were likely influenced by differences in clinical CS trajectories and center-specific referral practices and may also have been affected by limitations in available resources at the referring site. They emphasize the importance of structured referral pathways and heightened awareness at hub centers for this potentially high-risk IABP-treated CS cohort.

## Appendix

### Cardiogenic Shock Working Group Research Consortium:

Jonas Sundermeyer, MD^a,b^, Song Li, MD^c^, Van-Khue Ton, MD, PhD^d^, Rachna Kataria, MD^e^, Elric Zweck, MD, MPH^f^, Kevin J. John, MD^a^, Manreet K. Kanwar, MD^g^, Jaime Hernandez-Montfort, MD, MPH^h^, Shashank S. Sinha, MD, MSc^i^, A. Reshad Garan, MD^j^, Jacob Abraham, MD^k^, Vanessa Blumer, MD^l^, Ajar Kochar, MD^m^, Karthikeyan Ranganathan, MD^n^, Gavin W. Hickey MD^o^, Mohit Pahuja, MD^p^, Scott Lundgren, MD^q^, Sandeep Nathan MD^r^, Esther Vorovich, MD^s^, Shelley Hall, MD^t^, Wissam Khalife, MD^u^, Andrew Schwartzman, MD^v^, Ju Kim, MD^w^, Oleg Alec Vishnevsky, MD^x^, Justin Fried, MD^y^, Maryjane Farr, MD^z^, Joseph Mishkin, MD^aa^, I-Hui Chang, MD^bb^, Onyedika Ilonze, MD^cc^, Alexandra Arias, MD^dd^, Jun Nakata, MD^ee^, Jeffrey Marbach, MD^ff^, Hiram Bezerra, MD^gg^, Ann Gage, MD^hh^, Joyce Wald, MD^ii^, Sunu Thomas, MD^jj^, Faisal Rahman, MD^kk^, Amirali Masoumi, MD^ll^, Aasim Afzal, MD^mm^, Salman Gohar, MD^nn^, Rachel Goodman, MD^a^, Karol D. Walec, BS^a^, Peter S. Natov, MD^a^, Borui Li, MA^a^, Paavni Sangal, MPH^a^, Qiuyue Kong, MS^a^, Peter Zazzali, MS, MPH^a^, Neil M. Harwani, MS^a^, Saraschandra Vallabhajosyula, MD^oo^, Arvind Bhimaraj, MD^pp^, Claudius Mahr, MD^c^, Daniel Burkhoff, MD, PhD^qq^, Navin K. Kapur, MD^a^

^a^The Cardiovascular Center, Tufts Medical Center, Boston, Massachusetts; ^b^Department of Cardiology, University Heart and Vascular Center Hamburg, University Medical Center Hamburg-Eppendorf, Hamburg, Germany; ^c^Institute for Advanced Cardiac Care, Medical City Healthcare, Dallas, Texas; ^d^Massachusetts General Hospital, Boston, Massachusetts; ^e^Brown University Health Cardiovascular Institute, Providence, Rhode Island; ^f^Department of Cardiology, Pulmonology, and Vascular Medicine, Medical Faculty and University Hospital Düsseldorf, Heinrich-Heine-University, Düsseldorf, Germany; ^g^University of Chicago, Chicago, Illinois; ^h^Baylor Scott & White Health, Advanced Heart Failure Program Clinic, Temple, Texas; ^i^Inova Heart and Vascular Institute, Inova Fairfax Campus, Falls Church, Virginia; ^j^Beth Israel Deaconess Medical Center, Boston, Massachusetts; ^k^Center for Cardiovascular Analytics, Research, & Data Science (CARDS), Providence St. Joseph Research Network, Portland, Oregon; ^l^Inova Heart and Vascular Institute, Inova Fairfax Campus, Falls Church, Virginia; ^13m^Division of Cardiovascular Medicine, Brigham and Women’s Hospital, Boston, Massachusetts; ^n^Cardiovascular Institute at Allegheny Health Network, Pittsburgh, Pennsylvania; ^o^University of Pittsburgh Medical Center, Pittsburgh, Pennsylvania; ^p^University of Oklahoma Health Science Center, Oklahoma City, Oklahoma; ^17q^University of Nebraska Medical Center, Omaha, Nebraska; ^r^University of Chicago, Chicago, Illinois; ^s^Northwestern Medicine, Chicago, Illinois; ^t^Baylor University Medical Center, Dallas, Texas; ^u^University of Texas Medical Branch, Galveston, Texas; ^v^Maine Medical Center, Portland, Oregon; ^w^Houston Methodist Research Institute, Houston, Texas; ^x^Thomas Jefferson University Hospital, Philadelphia, Pennsylvania; ^y^Columbia University Irving Medical Center, New York, New York; ^z^UT Southwestern, Dallas, Texas; ^aa^Atrium Health Sanger Heart and Vascular Institute, Charlotte, North Carolina; ^bb^Banner University Medical Center, Phoenix, Arizona; ^cc^Indiana University School of Medicine, Indianapolis, Indiana; ^dd^Instituto Nacional de Cardiologia Ignacio Chavez, Mexico City, Mexico; ^ee^Nippon Medical School, Tokyo, Japan; ^ff^Oregon Health State University, Portland, Oregon; ^gg^Tampa General Hospital, Tampa, Florida; ^hh^TriStar Centennial Medical Center, Nashville, Tennessee; ^ii^University of Pennsylvania, Philadelphia, Pennsylvania; ^jj^University of Washington Medical Center, Seattle, Washington; ^kk^Johns Hopkins University, Baltimore, Maryland; ^ll^Atlantic Health System, Morristown, New Jersey; ^mm^Baylor Scott and White, Plano, Texas; ^nn^Baylor Scott and White, Fort Worth, Texas; ^oo^Brown University Health, Providence, Rhode Island; ^pp^Houston Methodist Hospital, Houston, Texas; ^qq^Cardiovascular Research Foundation, New York, New York

## Footnotes

To access the supplementary material accompanying this article, visit the online version of the *Journal of the Society for Cardiovascular Angiography & Interventions* at 10.1016/j.jscai.2026.104268.
