# Supplementary material for: Clinical Characteristics and Outcomes in Intra-aortic Balloon Pump–Supported Cardiogenic Shock Among Patients Transferred to Tertiary Care Centers
Source: J Soc Cardiovasc Angiogr Interv. 2026 Apr 7;5(5):104268. doi: 10.1016/j.jscai.2026.104268 (PMC13198140; doi:10.1016/j.jscai.2026.104268)
Supplement: Supplementary Data [file mmc1.docx]

***SUPPLEMENTARY APPENDIX***

***Original Research Manuscript:***

***Clinical Characteristics and Outcomes in IABP-Supported Cardiogenic Shock Among Patients Transferred to Tertiary Care Centers***

***Cardiogenic Shock Working Group Research Consortium:***

Jonas Sundermeyer, MD^1,2^; Song Li, MD^3^; Van-Khue Ton, MD, PhD^4^; Rachna Kataria, MD^5^; Elric Zweck, MD, MPH^6^; Kevin J. John, MD^1^; Manreet K. Kanwar, MD^7^; Jaime Hernandez-Montfort, MD, MPH^8^; Shashank S. Sinha, MD, MSc^9^; A. Reshad Garan, MD^10^; Jacob Abraham, MD^11^; Vanessa Blumer, MD^12^; Ajar Kochar, MD^13^; Karthikeyan Ranganathan, MD^14^; Gavin W. Hickey MD^15^; Mohit Pahuja, MD^16^; Scott Lundgren, MD^17^; Sandeep Nathan MD^18^; Esther Vorovich, MD^19^; Shelley Hall, MD^20^; Wissam Khalife, MD^21^; Andrew Schwartzman, MD^22^; Ju Kim, MD^23^; Oleg Alec Vishnevsky, MD^24^; Justin Fried, MD^25^; Maryjane Farr, MD^26^; Joseph Mishkin, MD^27^; I-Hui Chang, MD^28^; Onyedika Ilonze, MD^29^; Alexandra Arias, MD^30^; Jun Nakata, MD^31^; Jeffrey Marbach, MD^32^; Hiram Bezerra, MD^33^; Ann Gage, MD^34^; Joyce Wald, MD^35^; Sunu Thomas, MD^36^; Faisal Rahman, MD^37^; Amirali Masoumi, MD^38^; Aasim Afzal, MD^39^; Salman Gohar, MD^40^; Rachel Goodman, MD^1^; Karol D. Walec, BS^1^; Peter S. Natov, MD^1^; ﻿Borui Li, MA^1^; Paavni Sangal, MPH^1^; ﻿Qiuyue Kong, MS^1^; Peter Zazzali, MS, MPH^1^; Neil M. Harwani, MS^1^; Saraschandra Vallabhajosyula, MD^41^; Arvind Bhimaraj, MD^42^; Claudius Mahr, MD^3^;

Daniel Burkhoff, MD, PhD^43^; and Navin K. Kapur, MD^1^

1 The Cardiovascular Center, Tufts Medical Center, Boston, Massachusetts, USA

2 Department of Cardiology, University Heart and Vascular Center Hamburg, University Medical Center Hamburg-Eppendorf, Hamburg, Germany

3 Institute for Advanced Cardiac Care, Medical City Healthcare, Dallas, Texas, USA

4 Massachusetts General Hospital, Boston, USA

5 Brown University Health Cardiovascular Institute, Providence, Rhode Island

6 Department of Cardiology, Pulmonology, and Vascular Medicine, Medical Faculty and University Hospital Düsseldorf, Heinrich-Heine-University, Düsseldorf, Germany

7 University of Chicago, Chicago, USA

8 Baylor Scott & White Health, Advanced Heart Failure Program Clinic, Temple, USA

9 Inova Heart and Vascular Institute, Inova Fairfax Campus, Falls Church, USA

10 Beth Israel Deaconess Medical Center, Boston, USA

11 Center for Cardiovascular Analytics, Research, & Data Science (CARDS), Providence St. Joseph Research Network, Portland, USA

12 Inova Heart and Vascular Institute, Inova Fairfax Campus, Falls Church, VA

13 Division of Cardiovascular Medicine, Brigham and Women’s Hospital, Boston, USA

14 Cardiovascular Institute at Allegheny Health Network, Pittsburgh, USA

15 University of Pittsburgh Medical Center, Pittsburgh, USA

16 University of Oklahoma Health Science Center, Oklahoma City, USA

17 University of Nebraska Medical Center, Omaha, USA

18 University of Chicago, Chicago, USA

19 Northwestern Medicine, Chicago, USA

20 Baylor University Medical Center, Dallas, USA

21 University of Texas Medical Branch, Galveston, USA

22 Maine Medical Center, Portland, USA

23 Houston Methodist Research Institute, Houston, USA

24 Thomas Jefferson University Hospital, Philadelphia, USA

25 Columbia University Irving Medical Center, New York, USA

26 UT Southwestern, Dallas, USA

27 Atrium Health Sanger Heart and Vascular Institute, Charlotte, USA

28 Banner University Medical Center, Phoenix, USA
29 Indiana University School of Medicine, Indianapolis, USA

30 Instituto Nacional de Cardiologia Ignacio Chavez, Mexico City, Mexico

31 Nippon Medical School, Tokyo, Japan

32 Oregon Health State University, Portland, Oregon

33 Tampa General Hospital, Tampa, Florida, USA

34 TriStar Centennial Medical Center, Nashvil, USA

35 University of Pennsylvania, Philadelphia, USA

36 University of Washington Medical Center, Seattle, USA

37 Johns Hopkins University, Baltimore, USA

38 Atlantic Health System, Morristown, USA

39 Baylor Scott and White, Plano, USA

40 Baylor Scott and White, Fort Worth, USA

41 Brown University Health, Providence, USA

42 Houston Methodist Hospital, Houston, USA

43 Cardiovascular Research Foundation, New York, USA

***Supplementary Tables:***

- ***Supplemental Table S1****.* Characteristics for IABP-treated patients with AMI-CS stratified by transfer status.
- ***Supplemental Table S2.*** Laboratory and hemodynamic characteristics of IABP-treated patients with AMI-CS stratified by transfer status.
- ***Supplemental Table S3.*** Characteristics for IABP-treated patients with HF-CS stratified by transfer status.
- ***Supplemental Table S4.*** Laboratory and hemodynamic characteristics of IABP-treated patients with HF-CS stratified by transfer status.

***Supplementary Figures:***

- ***Supplemental Figure S1:*** Association between transfer status and transplant.
- ***Supplemental Figure S2:*** Association between transfer status and LVAD implantation.
- ***Supplemental Figure S3:*** Transfer status and in-hospital complications in AMI-CS, HF-CS, Non-AMI/Non-HF-CS.
- ***Supplemental Figure S4:*** Association between transfer status and in-hospital complications in AMI-CS.
- ***Supplemental Figure S5:*** Association between transfer status and in-hospital complications in HF-CS.
- ***Supplemental Figure S6:*** Association between transfer status and in-hospital complications in Non-AMI/Non-HF-CS.
- ***Supplemental Figure S7:*** Association between transfer status and use of vasoactive drugs.
- ***Supplemental Figure S8:*** Association between transfer status and use of mechanical ventilation.
- ***Supplemental Figure S9:*** Association between transfer status and ventilation duration.
- ***Supplemental Figure S10:*** Association between transfer status and use of RRT.

***Supplementary Tables***

***Supplemental Table S1****.* Characteristics for IABP-treated patients with AMI-CS stratified by transfer status.

| **Characteristic** | **Overall** | **Non-Transfer** | **Transfer** | **p-value** |
| --- | --- | --- | --- | --- |
|  | N = 772 | N = 395 | N = 377 |  |
| Sex |  |  |  | 0.009 |
| Female | 224 / 771 (29.1%) | 131 / 394 (33.2%) | 93 / 377 (24.7%) |  |
| Male | 547 / 771 (70.9%) | 263 / 394 (66.8%) | 284 / 377 (75.3%) |  |
| Age | 66 (59, 74) | 67 (61, 75) | 64 (57, 72) | <0.001 |
| Body Mass Index (kg/m²) | 27.7 (24.4, 31.8) | 27.2 (24.3, 30.7) | 28.4 (24.8, 32.5) | 0.031 |
| Hypertension | 548 / 768 (71.4%) | 291 / 392 (74.2%) | 257 / 376 (68.4%) | 0.071 |
| Diabetes mellitus | 362 / 767 (47.2%) | 196 / 391 (50.1%) | 166 / 376 (44.1%) | 0.10 |
| Atrial fibrillation/flutter | 91 / 767 (11.9%) | 53 / 391 (13.6%) | 38 / 376 (10.1%) | 0.14 |
| Chronic kidney disease | 145 / 767 (18.9%) | 92 / 391 (23.5%) | 53 / 376 (14.1%) | <0.001 |
| Peripheral vascular disease | 77 / 766 (10.1%) | 48 / 391 (12.3%) | 29 / 375 (7.7%) | 0.037 |
| COPD | 75 / 766 (9.8%) | 55 / 391 (14.1%) | 20 / 375 (5.3%) | <0.001 |
| Asthma | 35 / 737 (4.7%) | 19 / 379 (5.0%) | 16 / 358 (4.5%) | 0.7 |
| Cancer | 76 / 748 (10.2%) | 48 / 383 (12.5%) | 28 / 365 (7.7%) | 0.028 |
| Liver disease | 15 / 750 (2.0%) | 9 / 384 (2.3%) | 6 / 366 (1.6%) | 0.5 |
| Anemia | 101 / 739 (13.7%) | 63 / 380 (16.6%) | 38 / 359 (10.6%) | 0.018 |
| History of stroke/TIA | 83 / 767 (10.8%) | 54 / 391 (13.8%) | 29 / 376 (7.7%) | 0.007 |
| Severe valve disease | 85 / 768 (11.1%) | 57 / 392 (14.5%) | 28 / 376 (7.4%) | 0.002 |
| Prior CAD | 340 / 729 (46.6%) | 184 / 378 (48.7%) | 156 / 351 (44.4%) | 0.3 |
| History of HF | 226 / 765 (29.5%) | 124 / 391 (31.7%) | 102 / 374 (27.3%) | 0.2 |
| History of MI | 201 / 766 (26.2%) | 116 / 392 (29.6%) | 85 / 374 (22.7%) | 0.031 |
| Number of comorbidities | 3 (1, 5) | 3 (1, 5) | 2 (1, 4) | <0.001 |
| OHCA | 136 / 766 (17.8%) | 49 / 392 (12.5%) | 87 / 374 (23.3%) | <0.001 |
| LVEF baseline | 27.5 (20.0, 39.0) | 30.0 (21.0, 40.0) | 26.5 (18.0, 37.6) | 0.036 |
| SCAI B baseline | 64 / 518 (12.4%) | 59 / 212 (27.8%) | 5 / 306 (1.6%) | <0.001 |
| SCAI C baseline | 172 / 518 (33.2%) | 45 / 212 (21.2%) | 127 / 306 (41.5%) | <0.001 |
| SCAI D baseline | 141 / 518 (27.2%) | 58 / 212 (27.4%) | 83 / 306 (27.1%) | >0.9 |
| SCAI E baseline | 141 / 518 (27.2%) | 50 / 212 (23.6%) | 91 / 306 (29.7%) | 0.12 |
| SCAI B 24h | 1 / 377 (0.3%) | 1 / 192 (0.5%) | 0 / 185 (0.0%) | >0.9 |
| SCAI C 24h | 220 / 377 (58.4%) | 122 / 192 (63.5%) | 98 / 185 (53.0%) | 0.037 |
| SCAI D 24h | 86 / 377 (22.8%) | 44 / 192 (22.9%) | 42 / 185 (22.7%) | >0.9 |
| SCAI E 24h | 70 / 377 (18.6%) | 25 / 192 (13.0%) | 45 / 185 (24.3%) | 0.005 |
| SCAI B Max | 0 / 172 (0.0%) | 0 / 66 (0.0%) | 0 / 106 (0.0%) |  |
| SCAI C Max | 37 / 172 (21.5%) | 17 / 66 (25.8%) | 20 / 106 (18.9%) | 0.3 |
| SCAI D Max | 62 / 172 (36.0%) | 24 / 66 (36.4%) | 38 / 106 (35.8%) | >0.9 |
| SCAI E Max | 73 / 172 (42.4%) | 25 / 66 (37.9%) | 48 / 106 (45.3%) | 0.3 |
| VA-ECMO | 116 / 772 (15.0%) | 44 / 395 (11.1%) | 72 / 377 (19.1%) | 0.002 |
| Impella CP | 84 / 772 (10.9%) | 32 / 395 (8.1%) | 52 / 377 (13.8%) | 0.011 |
| Impella 5.0 | 3 / 772 (0.4%) | 3 / 395 (0.8%) | 0 / 377 (0.0%) | 0.2 |
| Impella 5.5 | 85 / 772 (11.0%) | 29 / 395 (7.3%) | 56 / 377 (14.9%) | <0.001 |
| IABP Access Site |  |  |  | <0.001 |
| Axillary | 24 / 442 (5.4%) | 15 / 392 (3.8%) | 9 / 50 (18.0%) |  |
| Femoral | 418 / 442 (94.6%) | 377 / 392 (96.2%) | 41 / 50 (82.0%) |  |
| Number of MCS Devices (during stay) | | |  | 0.4 |
| 2 | 714 / 772 (92.5%) | 362 / 395 (91.6%) | 352 / 377 (93.4%) |  |
| ≥3 | 58 / 772 (7.5%) | 33 / 395 (8.4%) | 25 / 377 (6.6%) |  |
| Number of max vasoactive drugs (during stay) | | |  | 0.3 |
| 0 | 27 / 432 (6.3%) | 15 / 218 (6.9%) | 12 / 214 (5.6%) |  |
| 1 | 108 / 432 (25.0%) | 58 / 218 (26.6%) | 50 / 214 (23.4%) |  |
| 2 | 118 / 432 (27.3%) | 65 / 218 (29.8%) | 53 / 214 (24.8%) |  |
| 3 | 114 / 432 (26.4%) | 53 / 218 (24.3%) | 61 / 214 (28.5%) |  |
| ≥4 | 65 / 432 (15.0%) | 27 / 218 (12.4%) | 38 / 214 (17.8%) |  |
| Mechanical Ventilation | 557 / 768 (72.5%) | 261 / 394 (66.2%) | 296 / 374 (79.1%) | <0.001 |
| RRT | 170 / 770 (22.1%) | 83 / 393 (21.1%) | 87 / 377 (23.1%) | 0.5 |
| Heart Transplant | 19 / 772 (2.5%) | 5 / 395 (1.3%) | 14 / 377 (3.7%) | 0.028 |
| LVAD Implantation | 91 / 770 (11.8%) | 37 / 394 (9.4%) | 54 / 376 (14.4%) | 0.033 |
| In-Hospital Mortality | 271 / 772 (35.1%) | 136 / 395 (34.4%) | 135 / 377 (35.8%) | 0.7 |
| 30-Day Mortality | 235 / 771 (30.5%) | 115 / 394 (29.2%) | 120 / 377 (31.8%) | 0.4 |
| Stroke During | 64 / 763 (8.4%) | 25 / 391 (6.4%) | 39 / 372 (10.5%) | 0.042 |
| In-Hospital Cardiac Arrest | 220 / 768 (28.6%) | 115 / 395 (29.1%) | 105 / 373 (28.2%) | 0.8 |
| Limb Ischemia | 70 / 763 (9.2%) | 26 / 390 (6.7%) | 44 / 373 (11.8%) | 0.014 |
| Acute Kidney Injury | 434 / 762 (57.0%) | 214 / 390 (54.9%) | 220 / 372 (59.1%) | 0.2 |
| Bleeding Requiring Surgery | 37 / 765 (4.8%) | 12 / 391 (3.1%) | 25 / 374 (6.7%) | 0.020 |
| Bleeding Requiring Transfusion | 187 / 758 (24.7%) | 86 / 386 (22.3%) | 101 / 372 (27.2%) | 0.12 |
| Hemolysis | 73 / 757 (9.6%) | 34 / 388 (8.8%) | 39 / 369 (10.6%) | 0.4 |

Binary variables are presented as absolute numbers and relative frequencies, and comparisons were conducted using Pearson’s Chi-squared test or Fisher’s exact test. Continuous variables are shown as the median with interquartile range (IQR) and were compared using the Wilcoxon rank-sum test.

CAD = coronary artery disease; COPD = chronic obstructive pulmonary disease; HF = heart failure; IABP = intra-aortic ballon pump; LVEF = left ventricular ejection fraction; MCS = mechanical circulatory support; MI = myocardial infarction; OHCA = out-of-hospital cardiac arrest; RRT = renal replacement therapy; SCAI = society for cardiovascular angiography and interventions; VA-ECMO = veno-arterial extracorporeal membrane oxygenation; TIA = transient ischemic attack.

***Supplemental Table S2.*** Laboratory and hemodynamic characteristics of IABP-treated patients with AMI-CS stratified by transfer status.

|  |  | **Transferred IABP-treated Patients** | | | **Non-transferred IABP-treated Patients** |  |
| --- | --- | --- | --- | --- | --- | --- |
| **Characteristic** | **Overall**  **(AMI-CS)** | **OSH Admission** | **OSH before Transfer** | **OSH Arrival Hub center** | **Baseline** | **p-value** |
|  | N = 772 | N = 377 | N = 377 | N = 377 | N = 395 |  |
| Lactate (mEq/L) | 2.6 (1.7, 4.8) | 3.3 (2.0, 6.4) | 2.4 (1.6, 4.6) | 1.8 (1.3, 3.7) | 2.4 (1.6, 4.5) | <0.001 |
| pH | 7.3 (7.2, 7.4) | 7.3 (7.2, 7.4) | 7.4 (7.3, 7.5) | 7.4 (7.3, 7.4) | 7.3 (7.3, 7.4) | <0.001 |
| ALT (IU/L) | 37.0 (20.0, 87.0) | 42.5 (21.0, 88.0) | 80.5 (37.0, 207.5) | 76.0 (37.0, 210.0) | 34.0 (19.0, 87.0) | <0.001 |
| AST (IU/L) | 62.0 (29.0, 198.0) | 56.0 (25.0, 205.0) | 171.0 (57.0, 409.0) | 178.0 (67.0, 442.0) | 64.0 (30.0, 198.0) | <0.001 |
| Serum Creatinine (mg/dL) | 1.3 (1.0, 1.8) | 1.3 (1.0, 1.7) | 1.3 (1.0, 2.0) | 1.3 (1.0, 2.0) | 1.3 (1.0, 1.9) | 0.5 |
| Systolic BP (mmHg) | 115.5 (100.0, 135.0) | 116.0 (100.0, 137.0) | 110.0 (98.0, 129.0) | 113.0 (97.0, 131.0) | 115.0 (100.0, 134.0) | 0.022 |
| Diastolic BP (mmHg) | 72.0 (62.0, 85.0) | 77.0 (59.0, 88.0) | 66.0 (54.5, 76.0) | 67.0 (54.0, 81.0) | 71.0 (62.0, 83.0) | <0.001 |
| Heart Rate (bpm) | 95.0 (78.0, 109.0) | 95.5 (78.5, 110.5) | 90.0 (74.0, 102.0) | 88.5 (75.0, 103.0) | 94.0 (78.0, 109.0) | <0.001 |
| MAP (mmHg) | 86.0 (75.0, 100.0) | 89.0 (72.0, 107.0) | 82.0 (70.0, 95.0) | 82.0 (72.5, 98.0) | 85.0 (75.0, 97.0) | 0.048 |
| RAP (mmHg) | 12.0 (9.0, 17.0) | 14.0 (9.0, 18.0) | 12.0 (9.0, 16.0) | 12.0 (9.0, 17.5) | 12.0 (9.0, 15.0) | 0.4 |
| PA Systolic Pressure (mmHg) | 43.0 (35.0, 53.0) | 43.5 (35.5, 51.5) | NA | 39.0 (31.0, 49.0) | 41.0 (34.0, 56.0) | 0.069 |
| PA Diastolic Pressure (mmHg) | 23.0 (19.0, 28.0) | 23.0 (19.0, 28.0) | 20.0 (15.0, 25.0) | 22.0 (17.0, 27.0) | 25.0 (22.0, 30.0) | 0.056 |
| PCWP (mmHg) | 23.5 (19.5, 28.0) | 23.5 (19.5, 28.0) | 24.0 (18.0, 28.0) | 24.0 (15.0, 30.0) | 23.0 (17.5, 29.0) | >0.9 |
| Cardiac Output (L/min) | 3.7 (2.9, 4.9) | 3.7 (3.1, 4.7) | 3.7 (3.0, 4.8) | 4.2 (3.2, 5.6) | 3.4 (2.6, 5.3) | 0.3 |

Data are presented as median with interquartile range. Group comparisons were performed using the Kruskal–Wallis test (overall p). ALT = alanine transaminase; AMI-CS = acute myocardial infarction-related cardiogenic shock; AST = aspartate transaminase; IABP = intra-aortic balloon pump; MAP = mean arterial pressure; OSH = outside hospital; RAP = right atrial pressure.

***Supplemental Table S3****.* Characteristics for IABP-treated patients with HF-CS stratified by transfer status.

| **Characteristic** | **Overall** | **Non-Transfer** | **Transfer** | **p-value** |
| --- | --- | --- | --- | --- |
|  | N = 1,032 | N = 853 | N = 179 |  |
| Sex |  |  |  | 0.8 |
| Female | 284 / 1,031 (27.5%) | 236 / 852 (27.7%) | 48 / 179 (26.8%) |  |
| Male | 747 / 1,031 (72.5%) | 616 / 852 (72.3%) | 131 / 179 (73.2%) |  |
| Age | 60 (50, 67) | 60 (50, 67) | 61 (53, 68) | 0.3 |
| Body Mass Index (kg/m²) | 27.8 (23.7, 32.1) | 27.7 (23.5, 32.0) | 28.0 (24.1, 32.7) | 0.3 |
| Hypertension | 671 / 1,029 (65.2%) | 554 / 850 (65.2%) | 117 / 179 (65.4%) | >0.9 |
| Diabetes mellitus | 431 / 1,030 (41.8%) | 358 / 851 (42.1%) | 73 / 179 (40.8%) | 0.8 |
| Atrial fibrillation/flutter | 435 / 1,031 (42.2%) | 368 / 852 (43.2%) | 67 / 179 (37.4%) | 0.2 |
| Chronic kidney disease | 412 / 1,032 (39.9%) | 363 / 853 (42.6%) | 49 / 179 (27.4%) | <0.001 |
| Peripheral vascular disease | 78 / 1,030 (7.6%) | 66 / 851 (7.8%) | 12 / 179 (6.7%) | 0.6 |
| COPD | 127 / 1,029 (12.3%) | 102 / 850 (12.0%) | 25 / 179 (14.0%) | 0.5 |
| Asthma | 92 / 1,002 (9.2%) | 81 / 828 (9.8%) | 11 / 174 (6.3%) | 0.2 |
| Cancer | 108 / 1,022 (10.6%) | 94 / 844 (11.1%) | 14 / 178 (7.9%) | 0.2 |
| Liver disease | 48 / 1,017 (4.7%) | 43 / 841 (5.1%) | 5 / 176 (2.8%) | 0.2 |
| Anemia | 234 / 1,012 (23.1%) | 198 / 838 (23.6%) | 36 / 174 (20.7%) | 0.4 |
| History of stroke/TIA | 158 / 1,032 (15.3%) | 133 / 853 (15.6%) | 25 / 179 (14.0%) | 0.6 |
| Severe valve disease | 277 / 1,032 (26.8%) | 238 / 853 (27.9%) | 39 / 179 (21.8%) | 0.093 |
| Prior CAD | 449 / 1,005 (44.7%) | 366 / 829 (44.1%) | 83 / 176 (47.2%) | 0.5 |
| History of HF | 883 / 1,031 (85.6%) | 760 / 853 (89.1%) | 123 / 178 (69.1%) | <0.001 |
| History of MI | 234 / 1,031 (22.7%) | 196 / 852 (23.0%) | 38 / 179 (21.2%) | 0.6 |
| Number of comorbidities | 4 (3, 6) | 4 (3, 6) | 4 (2, 5) | 0.008 |
| OHCA | 47 / 1,023 (4.6%) | 28 / 844 (3.3%) | 19 / 179 (10.6%) | <0.001 |
| SCAI B baseline | 147 / 636 (23.1%) | 146 / 479 (30.5%) | 1 / 157 (0.6%) | <0.001 |
| SCAI C baseline | 260 / 636 (40.9%) | 185 / 479 (38.6%) | 75 / 157 (47.8%) | 0.043 |
| SCAI D baseline | 156 / 636 (24.5%) | 105 / 479 (21.9%) | 51 / 157 (32.5%) | 0.008 |
| SCAI E baseline | 73 / 636 (11.5%) | 43 / 479 (9.0%) | 30 / 157 (19.1%) | <0.001 |
| SCAI B 24h | 14 / 450 (3.1%) | 11 / 367 (3.0%) | 3 / 83 (3.6%) | 0.7 |
| SCAI C 24h | 252 / 450 (56.0%) | 208 / 367 (56.7%) | 44 / 83 (53.0%) | 0.5 |
| SCAI D 24h | 134 / 450 (29.8%) | 112 / 367 (30.5%) | 22 / 83 (26.5%) | 0.5 |
| SCAI E 24h | 50 / 450 (11.1%) | 36 / 367 (9.8%) | 14 / 83 (16.9%) | 0.065 |
| SCAI B Max | 0 / 160 (0.0%) | 0 / 113 (0.0%) | 0 / 47 (0.0%) |  |
| SCAI C Max | 46 / 160 (28.8%) | 36 / 113 (31.9%) | 10 / 47 (21.3%) | 0.2 |
| SCAI D Max | 73 / 160 (45.6%) | 49 / 113 (43.4%) | 24 / 47 (51.1%) | 0.4 |
| SCAI E Max | 41 / 160 (25.6%) | 28 / 113 (24.8%) | 13 / 47 (27.7%) | 0.7 |
| VA-ECMO | 122 / 1,032 (11.8%) | 93 / 853 (10.9%) | 29 / 179 (16.2%) | 0.046 |
| Impella CP | 24 / 1,032 (2.3%) | 19 / 853 (2.2%) | 5 / 179 (2.8%) | 0.6 |
| Impella 2.5 | 1 / 1,032 (0.1%) | 1 / 853 (0.1%) | 0 / 179 (0.0%) | >0.9 |
| Impella 5.0 | 5 / 1,032 (0.5%) | 4 / 853 (0.5%) | 1 / 179 (0.6%) | >0.9 |
| Impella 5.5 | 235 / 1,032 (22.8%) | 196 / 853 (23.0%) | 39 / 179 (21.8%) | 0.7 |
| IABP Access Site |  |  |  | <0.001 |
| Axillary | 193 / 875 (22.1%) | 177 / 844 (21.0%) | 16 / 31 (51.6%) |  |
| Femoral | 682 / 875 (77.9%) | 667 / 844 (79.0%) | 15 / 31 (48.4%) |  |
| Number of MCS (during stay) | | |  | <0.001 |
| 2 | 873 / 1,032 (84.6%) | 701 / 853 (82.2%) | 172 / 179 (96.1%) |  |
| ≥3 | 159 / 1,032 (15.4%) | 152 / 853 (17.8%) | 7 / 179 (3.9%) |  |
| Number of max vasoactive drugs (during stay) | | |  | 0.015 |
| 0 | 22 / 610 (3.6%) | 14 / 526 (2.7%) | 8 / 84 (9.5%) |  |
| 1 | 195 / 610 (32.0%) | 168 / 526 (31.9%) | 27 / 84 (32.1%) |  |
| 2 | 186 / 610 (30.5%) | 169 / 526 (32.1%) | 17 / 84 (20.2%) |  |
| 3 | 121 / 610 (19.8%) | 101 / 526 (19.2%) | 20 / 84 (23.8%) |  |
| ≥4 | 86 / 610 (14.1%) | 74 / 526 (14.1%) | 12 / 84 (14.3%) |  |
| Mechanical Ventilation Stay | 796 / 1,023 (77.8%) | 664 / 844 (78.7%) | 132 / 179 (73.7%) | 0.15 |
| RRT Dialysis Stay | 234 / 1,029 (22.7%) | 205 / 850 (24.1%) | 29 / 179 (16.2%) | 0.022 |
| Heart Transplant | 314 / 1,031.0 (30.5%) | 277 / 852 (32.5%) | 37 / 179 (20.7%) | 0.002 |
| LVAD Implantation | 267 / 1,026 (26.0%) | 228 / 847 (26.9%) | 39 / 179 (21.8%) | 0.2 |
| In-Hospital Mortality | 232 / 1,032 (22.5%) | 190 / 853 (22.3%) | 42 / 179 (23.5%) | 0.7 |
| 30-Day Mortality | 166 / 1,029 (16.1%) | 133 / 851 (15.6%) | 33 / 178 (18.5%) | 0.3 |
| Stroke During Stay | 55 / 1,028 (5.4%) | 42 / 849 (4.9%) | 13 / 179 (7.3%) | 0.2 |
| In-Hospital Cardiac Arrest | 104 / 1,025 (10.1%) | 84 / 847 (9.9%) | 20 / 178 (11.2%) | 0.6 |
| Limb Ischemia | 36 / 1,026 (3.5%) | 27 / 847 (3.2%) | 9 / 179 (5.0%) | 0.2 |
| Acute Kidney Injury | 674 / 1,021 (66.0%) | 558 / 843 (66.2%) | 116 / 178 (65.2%) | 0.8 |
| Bleeding Requiring Surgery | 60 / 1,027 (5.8%) | 53 / 848 (6.3%) | 7 / 179 (3.9%) | 0.2 |
| Bleeding Requiring Transfusion | 222 / 1,013 (21.9%) | 183 / 838 (21.8%) | 39 / 175 (22.3%) | 0.9 |
| Hemolysis | 91 / 1,010 (9.0%) | 77 / 832 (9.3%) | 14 / 178 (7.9%) | 0.6 |

Binary variables are presented as absolute numbers and relative frequencies, and comparisons were conducted using Pearson’s Chi-squared test or Fisher’s exact test. Continuous variables are shown as the median with interquartile range (IQR) and were compared using the Wilcoxon rank-sum test.

CAD = coronary artery disease; COPD = chronic obstructive pulmonary disease; HF = heart failure; IABP = intra-aortic ballon pump; LVEF = left ventricular ejection fraction; MCS = mechanical circulatory support; MI = myocardial infarction; OHCA = out-of-hospital cardiac arrest; RRT = renal replacement therapy; SCAI = society for cardiovascular angiography and interventions; VA-ECMO = veno-arterial extracorporeal membrane oxygenation; TIA = transient ischemic attack.

***Supplemental Table S4****.* Laboratory and hemodynamic characteristics of IABP-treated patients with HF-CS stratified by transfer status.

|  |  | **Transferred IABP-treated Patients** | | | **Non-transferred IABP-treated Patients** |  |
| --- | --- | --- | --- | --- | --- | --- |
| **Characteristic** | **Overall (HF-CS)** | **OSH Admission** | **OSH before Transfer** | **OSH Arrival Hub Center** | **Admission** | **p-value** |
|  | N = N = 1,032 | N = 179 | N = 179 | N = 179 | N = 853 |  |
| Lactate (mEq/L) | 1.9 (1.4, 3.0) | 2.8 (1.8, 4.7) | 2.2 (1.4, 3.7) | 1.4 (1.1, 2.2) | 1.9 (1.4, 2.8) | <0.001 |
| pH | 7.4 (7.3, 7.5) | 7.4 (7.3, 7.4) | 7.4 (7.4, 7.4) | 7.4 (7.4, 7.5) | 7.4 (7.4, 7.5) | 0.007 |
| ALT (IU/L) | 36.0 (19.0, 91.0) | 49.0 (23.0, 159.0) | 111.0 (25.0, 665.0) | 73.0 (22.0, 462.0) | 34.0 (18.0, 84.5) | <0.001 |
| AST (IU/L) | 36.0 (23.0, 81.0) | 53.5 (25.0, 193.0) | 86.0 (30.0, 532.0) | 64.0 (26.0, 386.0) | 35.0 (23.0, 77.0) | <0.001 |
| Serum Creatinine (mg/dL) | 1.5 (1.1, 2.1) | 1.5 (1.2, 2.0) | 1.5 (1.1, 2.1) | 1.5 (1.1, 2.0) | 1.5 (1.1, 2.1) | 0.8 |
| Systolic BP (mmHg) | 105.0 (95.0, 118.0) | 110.0 (94.0, 122.0) | 110.0 (97.0, 124.0) | 112.0 (97.0, 127.0) | 105.0 (95.0, 117.0) | 0.003 |
| Diastolic BP (mmHg) | 71.0 (62.0, 80.0) | 72.0 (61.0, 81.0) | 65.0 (53.0, 74.0) | 63.0 (53.0, 75.5) | 71.0 (63.0, 80.0) | <0.001 |
| Heart Rate (bpm) | 93.0 (79.0, 108.0) | 96.0 (81.0, 112.0) | 91.0 (77.5, 107.0) | 88.0 (78.0, 101.0) | 92.5 (79.0, 107.0) | 0.038 |
| MAP (mmHg) | 82.0 (74.0, 92.0) | 86.5 (72.0, 97.5) | 80.0 (69.0, 90.0) | 82.0 (73.0, 92.0) | 82.0 (74.0, 92.0) | 0.2 |
| RAP (mmHg) | 14.0 (10.0, 19.0) | 15.0 (11.0, 19.0) | 13.0 (8.0, 19.5) | 13.0 (9.0, 17.0) | 14.0 (8.0, 17.0) | 0.2 |
| PA Systolic Pressure (mmHg) | 46.0 (40.0, 60.0) | 49.0 (40.0, 60.0) | NA (NA, NA) | 46.0 (36.0, 53.0) | 45.0 (41.0, 61.0) | 0.14 |
| PA Diastolic Pressure (mmHg) | 27.0 (20.0, 34.0) | 28.0 (20.0, 34.5) | 24.0 (18.0, 30.0) | 24.0 (19.0, 29.0) | 26.0 (20.0, 32.0) | 0.076 |
| PCWP (mmHg) | 26.0 (20.0, 34.0) | 26.0 (20.0, 35.0) | 25.0 (20.0, 34.0) | 18.0 (16.0, 27.0) | 25.0 (20.0, 31.0) | 0.012 |
| Cardiac Output (L/min) | 3.3 (2.5, 4.4) | 3.0 (2.2, 4.1) | 3.7 (2.9, 4.9) | 3.9 (3.0, 5.1) | 3.6 (2.6, 4.5) | 0.018 |

Data are presented as median with interquartile range. Group comparisons were performed using the Kruskal–Wallis test (overall p). ALT = alanine transaminase; HF-CS = heart failure-related cardiogenic shock; AST = aspartate transaminase; IABP = intra-aortic balloon pump; MAP = mean arterial pressure; OSH = outside hospital; RAP = right atrial pressure.

***Supplementary Figures:***

***Supplemental Figure S1:*** Association between transfer status and transplant.

***
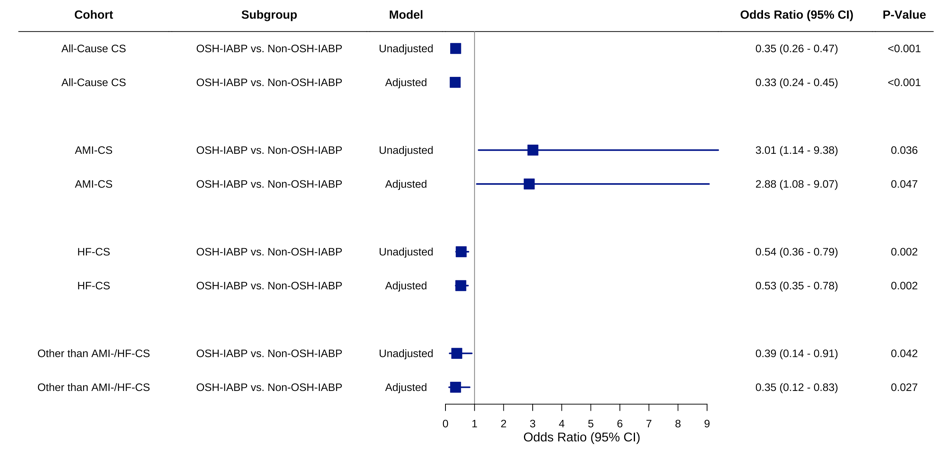
***Association between transfer status (OSH-IABP vs. Non-OSH-IABP) and heart transplant across cardiogenic shock subtypes. Odds ratios for transplant were calculated using multivariable logistic regression models in all-cause, acute myocardial infarction-related, heart failure-related, and non-acute myocardial infarction-/non–heart failure-related cardiogenic shock, adjusted for age and sex. AMI-CS = acute myocardial infarction-related cardiogenic shock; HF-CS = heart failure-related cardiogenic shock; IABP = intra-aortic balloon pump; OSH = outside hospital.

***Supplemental Figure S2:*** Association between transfer status and LVAD implantation.

***
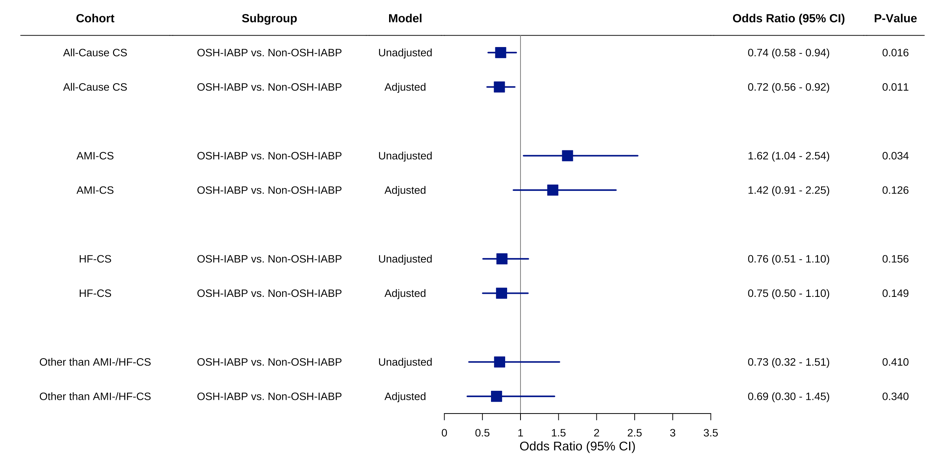
***

Association between transfer status (OSH-IABP vs. Non-OSH-IABP) and LVAD implantation across cardiogenic shock subtypes. Odds ratios for LVAD implantation were calculated using multivariable logistic regression models in all-cause, acute myocardial infarction-related, heart failure-related, and non-acute myocardial infarction-/non–heart failure-related cardiogenic shock, adjusted for age and sex. AMI-CS = acute myocardial infarction-related cardiogenic shock; HF-CS = heart failure-related cardiogenic shock; IABP = intra-aortic balloon pump; OSH = outside hospital.

***Supplemental Figure S3****:* Transfer status and in-hospital complications in AMI-CS, HF-CS, Non-AMI/Non-HF-CS.

***
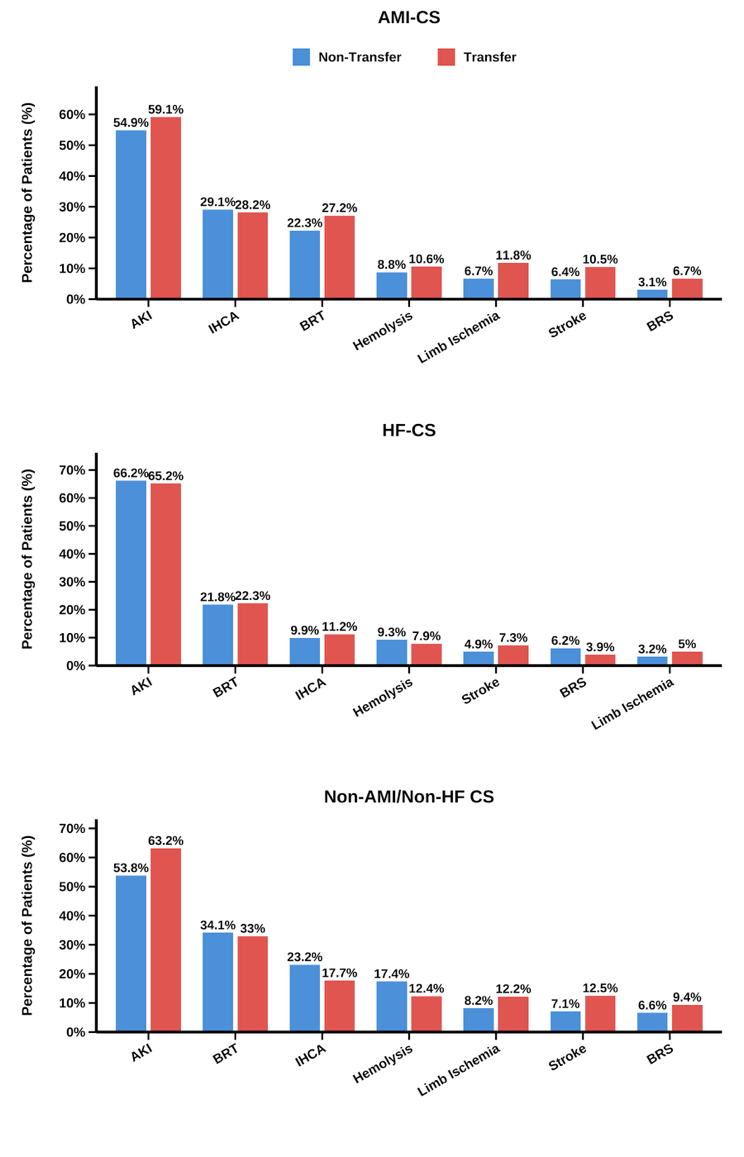
***

Crude incidence of in-hospital complications in IABP-treated patients stratified by transfer status in acute myocardial infarction-related (**A**), heart failure-related (**B**), and non-acute myocardial infarction-/non–heart failure-related cardiogenic shock (**C**). AMI-CS = acute myocardial infarction-related cardiogenic shock; AKI = acute kidney injury; HF-CS = heart failure-related cardiogenic shock; BRS = bleeding requiring surgery; BRT = bleeding requiring transfusion; IHCA = in-hospital cardiac arrest.

***Supplemental Figure S4:*** Association between transfer status and in-hospital complications in AMI-CS.

***
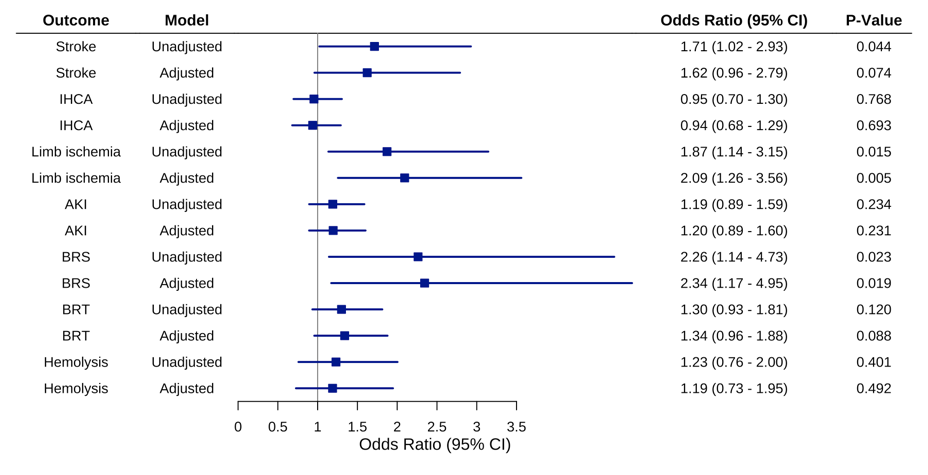
***

Odds ratios for complications associated with transfer status (transfer vs. non-transfer), using logistic regression models, adjusted for age and sex, in acute myocardial-infarction-related cardiogenic shock (AMI-CS). AKI = acute kidney injury; BRS = bleeding requiring surgery; BRT = bleeding requiring transfusion; IHCA = in-hospital cardiac arrest.

***Supplemental Figure S5:*** Association between transfer status and in-hospital complications in HF-CS.***
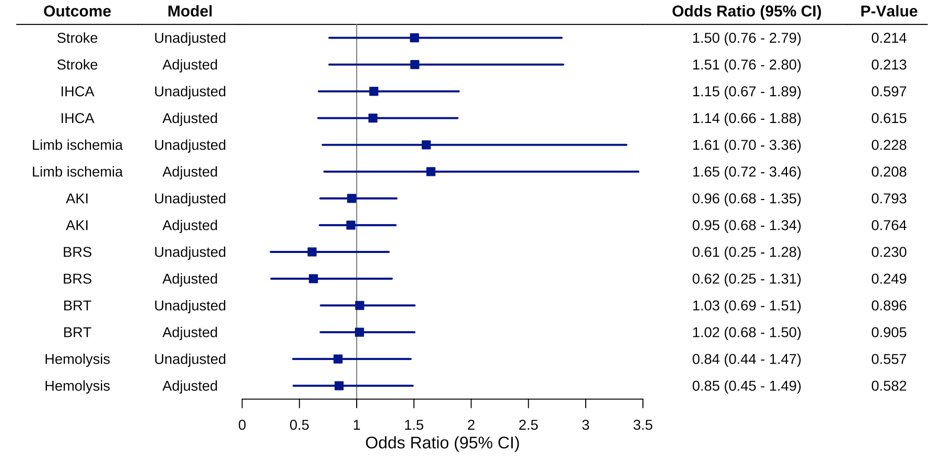
***Odds ratios for complications associated with transfer status (transfer vs. non-transfer), using logistic regression models, adjusted for age and sex, in heart failure-related cardiogenic shock (HF-CS). AKI = acute kidney injury; BRS = bleeding requiring surgery; BRT = bleeding requiring transfusion; IHCA = in-hospital cardiac arrest.

***Supplemental Figure S6:*** Association between transfer status and in-hospital complications in Non-AMI/Non-HF-CS.

***
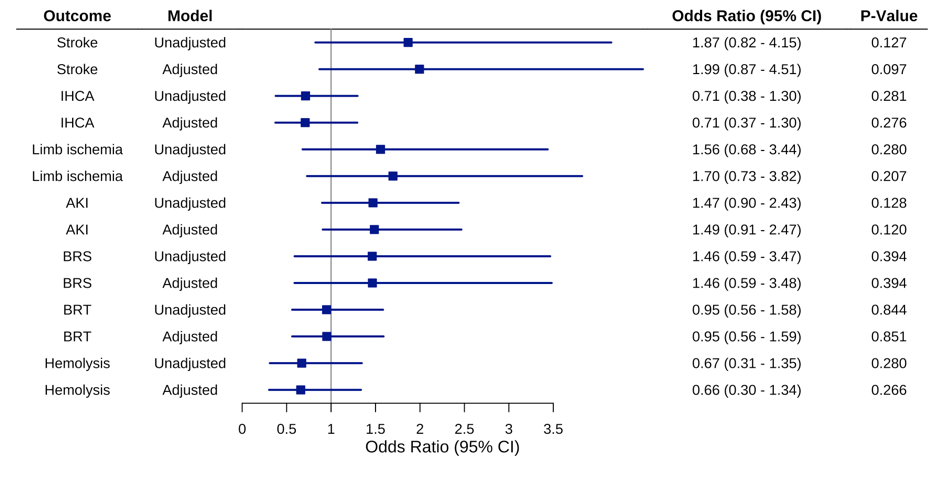
***

Odds ratios for complications associated with transfer status (transfer vs. non-transfer), using logistic regression models, adjusted for age and sex, in Non-AMI/Non-HF-CS. AKI = acute kidney injury; BRS = bleeding requiring surgery; BRT = bleeding requiring transfusion; IHCA = in-hospital cardiac arrest.

***Supplemental Figure S7:*** Association between transfer status and use of vasoactive drugs.

***
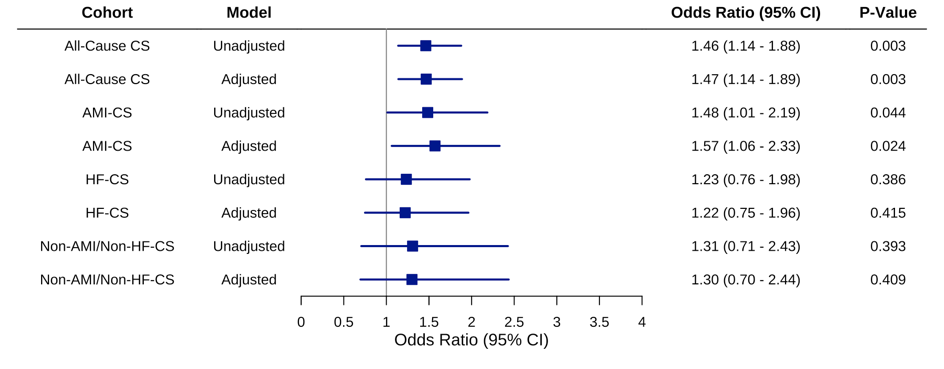
***

Association between transfer status (transfer vs. non-transfer) and the use of vasoactive drugs (≥2 vs. <2) across cardiogenic shock subtypes, based on logistic regression models, adjusted for age and sex. In all-cause and AMI-CS, transfer status was significantly associated with a higher likelihood of needing ≥2 vasoactive drugs. AMI-CS = acute myocardial infarction-related cardiogenic shock; HF-CS = heart failure-related cardiogenic shock.

***Supplemental Figure S8:*** Association between transfer status and use of mechanical ventilation.

***
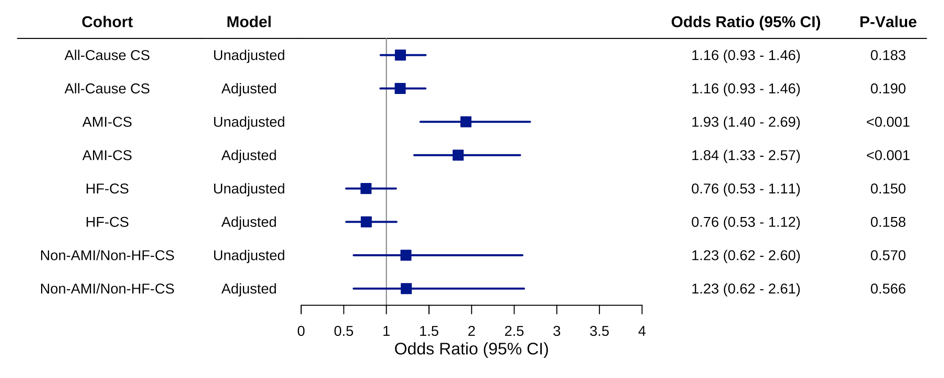
***

Association between transfer status (transfer vs. non-transfer) and the use of mechanical ventilation (yes vs. no) across cardiogenic shock subtypes, based on logistic regression models, adjusted for age and sex. AMI-CS = acute myocardial infarction-related cardiogenic shock; HF-CS = heart failure-related cardiogenic shock.

***Supplemental Figure S9:*** Association between transfer status and ventilation duration.***
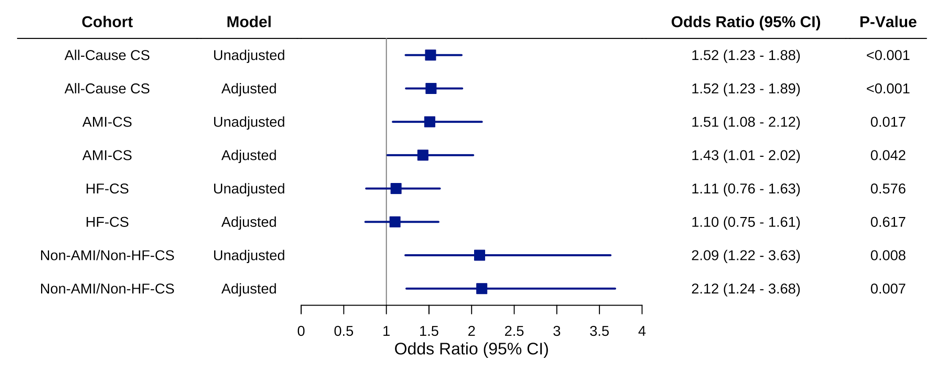
***

Association between transfer status (transfer vs. non-transfer) and the mechanical ventilation duration (days, >median vs. ≤median) across cardiogenic shock subtypes, based on logistic regression models, adjusted for age and sex. AMI-CS = acute myocardial infarction-related cardiogenic shock; HF-CS = heart failure-related cardiogenic shock.

***Supplemental Figure S10:*** Association between transfer status and use of RRT.

***
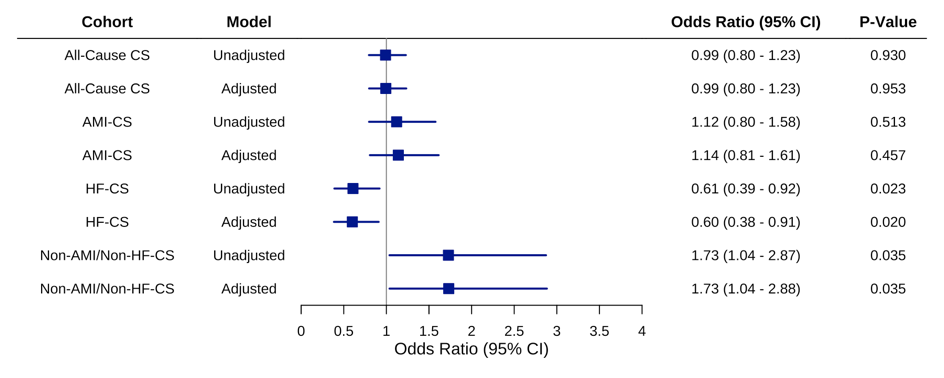
***

Association between transfer status (transfer vs. non-transfer) and the use of renal replacement therapy (RRT yes vs. RRT no) across cardiogenic shock subtypes, based on logistic regression models, adjusted for age and sex. AMI-CS = acute myocardial infarction-related cardiogenic shock; HF-CS = heart failure-related cardiogenic shock.
